# Computational prediction of workability and mechanical properties of bentonite plastic concrete using multi-expression programming

**DOI:** 10.1038/s41598-024-56088-0

**Published:** 2024-03-13

**Authors:** Majid Khan, Mujahid Ali, Taoufik Najeh, Yaser Gamil

**Affiliations:** 1 Department of Civil Engineering, COMSATS University Islamabad, Abbottabad Campus, Abbottabad, 22060 Pakistan; 2 Department of Transport Systems, Traffic Engineering and Logistics, Faculty of Transport and Aviation Engineering, Silesian University of Technology, Krasińskiego 8 Street, 40-019 Katowice, Poland; 3 Operation, Maintenance, and Acoustics, Department of Civil, Environmental and Natural Resources Engineering, Lulea University of Technology, Luleå, Sweden; 4 Department of Civil Engineering, School of Engineering, Monash University Malaysia, Jalan Lagoon Selatan, 47500 Bandar Sunway, Selangor Malaysia

**Keywords:** Bentonite plastic concrete, MEP, Machine learning, Slump, Mechanical properties, Engineering, Materials science

## Abstract

Bentonite plastic concrete (BPC) demonstrated promising potential for remedial cut-off wall construction to mitigate dam seepage, as it fulfills essential criteria for strength, stiffness, and permeability. High workability and consistency are essential attributes for BPC because it is poured into trenches using a tremie pipe, emphasizing the importance of accurately predicting the slump of BPC. In addition, prediction models offer valuable tools to estimate various strength parameters, enabling adjustments to BPC mixing designs to optimize project construction, leading to cost and time savings. Therefore, this study explores the multi-expression programming (MEP) technique to predict the key characteristics of BPC, such as slump, compressive strength (*fc*), and elastic modulus (*Ec*). In the present study, 158, 169, and 111 data points were collected from the experimental studies for the slump, *fc*, and *Ec,* respectively. The dataset was divided into three sets: 70% for training, 15% for testing, and another 15% for model validation. The MEP models exhibited excellent accuracy with a correlation coefficient (R) of 0.9999 for slump, 0.9831 for *fc,* and 0.9300 for *Ec.* Furthermore, the comparative analysis between MEP models and conventional linear and non-linear regression models revealed remarkable precision in the predictions of the proposed MEP models, surpassing the accuracy of traditional regression methods. SHapley Additive exPlanation analysis indicated that water, cement, and bentonite exert significant influence on slump, with water having the greatest impact on compressive strength, while curing time and cement exhibit a higher influence on elastic modulus. In summary, the application of machine learning algorithms offers the capability to deliver prompt and precise early estimates of BPC properties, thus optimizing the efficiency of construction and design processes.

## Introduction

The ageing infrastructure worldwide poses a significant concern for many nations. Unfortunately, public awareness regarding this issue tends to escalate only following a catastrophic failure in some aspect of the infrastructure^1^. For instance, during the Katrina and Rita Hurricanes in the Gulf Coast, embankment dams and levees experienced severe and widespread failure in 2005^2^. Earthen dams can fail in different ways, including insufficient maintenance, over-topping, foundation issues, and slope instability. The latter often happens when water seepage beneath the dam weakens internal friction, leading to the dam sliding or slipping^3^. As a result, significant attention has been directed towards ensuring the safety of dams, leading to the implementation of various global programs focused on dam repair and remediation^1^. A widely used approach to address dam seepage involves the construction of cut-off walls. There are numerous options for backfill materials in cut-off walls, but there is a growing interest in plastic concrete^4,5^. This is because of its favorable qualities, such as its elastic–plastic properties, low permeability, and homogeneity^6^.

Due to its excellent low permeability characteristics, bentonite is utilized to prepare plastic concrete to construct cut-off walls beneath dams to block water penetration^7^. Plastic concrete must possess robust strength, impermeability, and stiffness similar to the surrounding soil. Ensuring compatibility of strain between adjacent soil and the wall helps mitigate the risk of wall over-stressing and allows for deformation without separation^8^. This type of concrete holds significant potential in meeting the criteria for strength, stiffness, and permeability in the construction of remedial cut-off walls^7^. Although it offers enhanced formability, its strength is comparatively lower due to the incorporation of clay slurry^9^. Typically, plastic concrete includes typical concrete constituents and bentonite clay, and a greater water-binder ratio to yield a more workable and elastic material^9^. It is noteworthy that bentonite has long been employed for sealing purposes in hydraulic and civil structures^10–13^.

Bentonite plastic concrete (BPC) must have excellent workability and consistency because fresh concrete deposited into a trench by pipe must be capable of moving in the ditch and forcing the already poured concrete with high pressure^14^. This emphasizes the significance of forecasting the slump of BPC. Moreover, regulating seepage content and, ensuring the stability of dams is significantly influenced by the compressive strength (*fc*) of the employed plastic concrete (PC). Therefore, obtaining comprehensive details about factors impacting the *fc* of PC, including the mixing ratio and curing duration, is essential^14^. Numerous factors can impact the strength of BPC, including the attributes of concrete constituents, curing time, and mixing ratio. In dam construction sites and during the manufacturing of BPC, it is customary to subject samples from different mixers to testing using specialized equipment and expert personnel. This process is essential for ensuring quality control and reliability.^15,16^. However, challenges arise in the workplace, such as construction issues, storage, and curing processes for a large number of concrete samples^17–21^. The need for a prompt assessment of sample resistance to adjust ratios adds complexity and incurs significant time and costs. Therefore, having a reasonably accurate and comprehensive estimate of compressive strength (within the desired confidence level) is essential for making informed decisions^22,23^. Researchers have employed empirical regression methods to estimate the strength of BPC^24–26^.

In the past few decades, machine learning (ML) has garnered significant interest in its application to construction materials^18,27,28^. ML techniques, like neural network (NN) prediction models, were chosen from the beginning of the application of data mining^29^^30,31^. However, over time, alternative techniques such as adaptive probabilistic neural networks (APNN)^32^, fuzzy polynomial neural networks (FPNN)^33,34^, and GMDH-type neural networks^35,36^, were developed to improve the reliability, pace, and enhancing the performance of NN, but the artificial neural network (ANN) technique still holds the majority of literature^37–42^. In addition to ANN, several authors have employed other ML techniques in their studies, such as SVM and ANFIS^43–45^^46^. Nevertheless, the use of the ANN approach has certain drawbacks and limitations in prediction modeling^47–50^. To begin with, the ANN is categorized as a black-box approach, offering limited interpretation in terms of how the model generates its estimations^51–53^. The absence of clarity of interpretation may hinder understanding and confidence in the model, particularly in vital applications where interpretability holds significant importance. For example, Ekanayake et al.^54^ highlighted the difficulty faced by individuals lacking familiarity with ML methods in understanding them, often perceiving them as an enigmatic “black-box” approach^55–57^. The absence of vital information like the relationship between outputs and inputs, and the logic behind estimations, erodes end-users' trust in ML estimations^58^. In addition, ANN is susceptible to overfitting or underfitting the data. Overfitting occurs when the model becomes excessively complex, memorizing the training data and subsequently exhibiting poor generalization performance when applied to new, unseen data^59^. Moreover, fine-tuning hyperparameters in ANN models is frequently necessary to improve model performance. Identifying the ideal setup can pose challenges and may necessitate extensive experimentation through trial and error^60–63^. To address these issues, evolutionary algorithms (EAs) and genetic algorithms (GA), which include gene expression programming (GEP) and multi-expression programming (MEP), are being utilized to forecast concrete properties^49,50,53,57,64^. The superiority of such algorithms is the generation of useful mathematical expressions, as well as their great reliability and predictive potential.

Recently, few studies have been conducted to forecast the characteristics of BPC. For instance, Ghanizadehe et al.^14^ utilized ANN and SVM approaches to estimate the *fc* of BPC. Similarly, another study by Amlashi et al.^65^ employed four techniques (SVM, RSM, GMDH, MGGP) to forecast the *fc* of BPC. It was reported that the SVM model outperformed the remaining three models. Amlashi et al.^66^ also used SVM and adaptive Neuro-fuzzy inference system (ANFIS) methods optimized with particle swarm optimization (PSO) to estimate the *fc* of BPC. The majority of these studies focused on neural network methods, which lack transparency and interpretability aspects of ML modeling. Moreover, ANN methods are vulnerable to the issue of overfitting^67–69^.

To address the shortcomings of other neural algorithms, a novel approach known as MEP has been developed^51,70^. Due to the linear nature of chromosomes and their potential for coding several solutions on just one chromosome. The finest of the chosen chromosomes is selected as the final replica. In comparison to EA, MEP is an improved version of GP that can compute an accurate output even when the complexity of the objective is unknown. Contrary to ML techniques, MEP does not need the final equation's formulation to be determined. The mathematical discrepancies are determined and removed from the final formulation throughout the MEP development process. Furthermore, in comparison to other soft computing systems, the decoding procedure in MEP is significantly simpler. Despite the numerous advantages of MEP over other evolutionary algorithms, its utilization till now is limited in construction materials research. MEP was used by Alavi et al.^71^ to forecast soil classification established on the liquid limit (LL), plastic limit (PL), and soil color. Similarly, MEP is used for Marshall mix design, flow, and stability^72–74^.

In the present study, *fc*, elastic modulus (*Ec*), and slump of BPC have been modeled using the MEP technique while taking into consideration the most influential input variables. An extensive database has been collected and categorized into different sets (training, validation, testing) to guarantee that the model is effectively prepared. To ensure model applicability and accuracy, extensive statistical and performance checks are performed to measure model efficiency. In addition, SHAP analysis was used for the interpretability of the suggested models.

## Research methodology

### Multi-expression programming (MEP)

**Figure 1:**
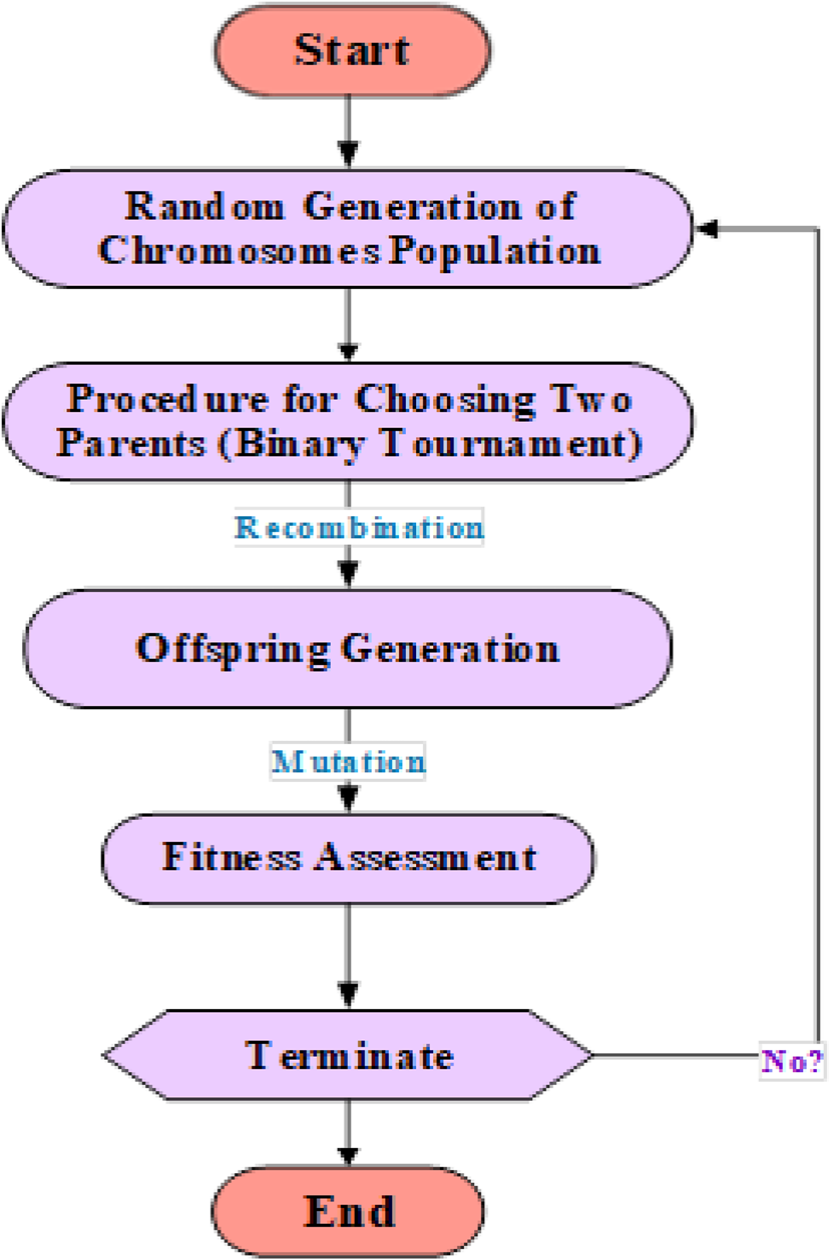
Flowchart illustration of MEP algorithm.

**Figure 2:**
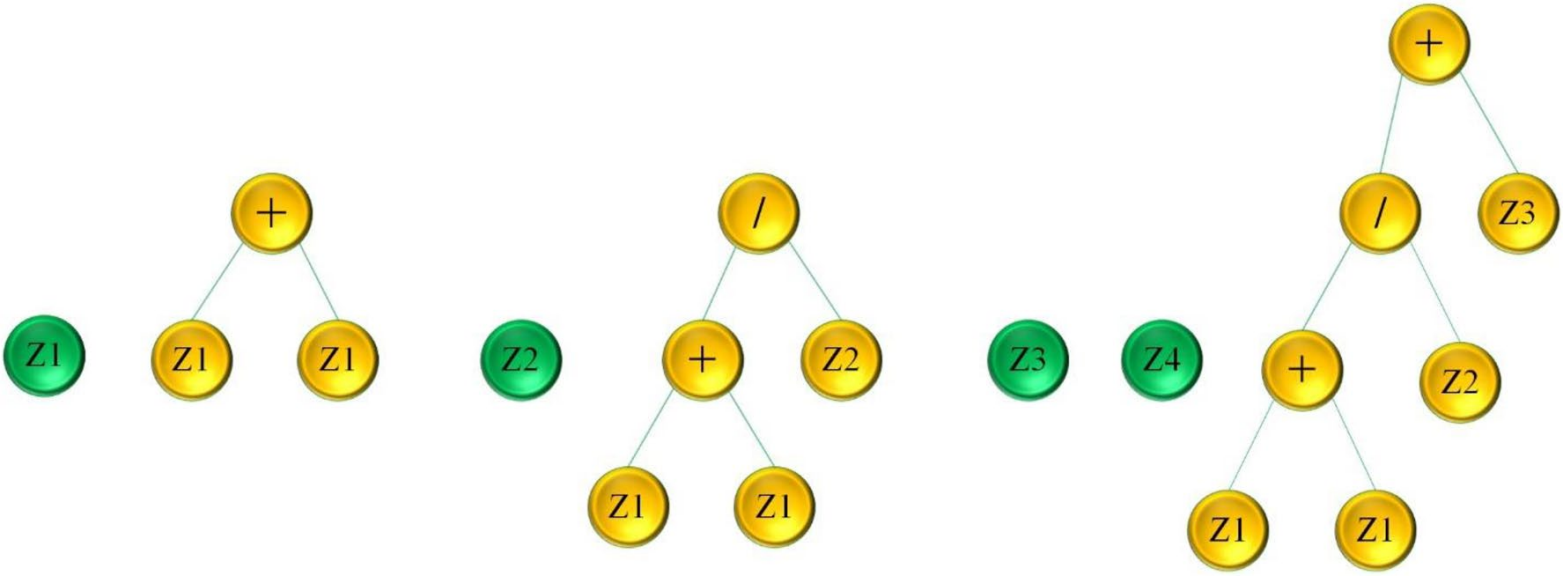
Architecture of MEP.

The objective of this modeling technique is to offer precise and useful mathematical formulations to predict output using pre-defined parameters. Koza^75^ introduced an extension of GA called GP, which is relying on Darwinian principles^14^. The fundamental distinction between both methods is that in GA, binary strings are used, but in GP, parse trees are used. Recently, multiple kinds of EAs have been suggested, with one of their main differences being linearity^75^. One method for describing the output of an MEP modeling is a linear string of commands with variables or operations. Figure 1 depicts the processes that occur in MEP development. The MEP algorithm forms through several stages: initially, it creates a diverse population of chromosomes. Then, it employs a binary tournament operation to select parents. With a constant crossover probability, it merges selected parents to produce offspring. Mutation introduces variation, and finally, the algorithm replaces inferior members of the population with the best-performing ones^51^. The process is iterative and continues until it reaches convergence^71^. Figure 2 depicts the MEP architecture.

MEP offers various advantages over other types of genetic techniques like genetic programming. GP uses a tree crossover evolutionary process, which produces several parse trees, increasing computational time and the need for storage^76^. In addition, since GP is both a phenotype and a genotype, it is difficult to provide a simple formulation for the required task. MEP maintains a large variety of expressions, including certain implicit structures, which is referred to as implicit parallelism. MEP also has the capacity to maintain many solutions to a problem on a single chromosome^51,70^. MEP can distinguish between phenotype and genotype due to the linear variations^74^. MEP is thought to be more effective than other ML methods due to its capacity to encode several answers inside a single chromosome. This unique feature enables MEP to look over for a better feasible response. Unlike other GP algorithms, MEP provides simple decoding operations and pays particular attention to cases where the specifics of the desired expression are unclears^51^. MEP can manage issues such as division by zero, improper expressions, and many more^77^. Furthermore, multi-gene genetic programming (MGGP) and MEP are both extensions of traditional GP designed to address complex optimization problems. While they share similarities in their approach, there are distinct differences in how they represent and evolve solutions. In MGGP, an individual is represented as a set of multiple genes, each of which may encode a distinct subcomponent or module of the solution^78,79^. These genes can be trees or other structures suitable for the problem domain. In MEP, an individual is represented as a set of multiple expressions, typically in the form of linear or matrix-based representations^80^. Each expression contributes to the overall solution and can be evaluated independently. Moreover, MGGP typically uses genetic operators such as crossover and mutation at the gene level^81^. It means that crossover and mutation operations can occur within individual genes, allowing for the exchange or modification of entire subcomponents of the solution. In contrast, MEP often employs mutation operators that act at the expression level, modifying individual expressions or parts of expressions to create new candidate solutions. Crossover operations in MEP may involve combining entire expressions from different individuals^82^.

### Experimental database

An extensive database of BPC has been collected from the existing literature for GEP modeling (provided in supplementary as Tables S1–S3)^83^. The database contains 158, 169, and 111 datasets for the slump, *fc*, and *Ec*, respectively. It must be noted that the samples used in experimental studies were of two distinct dimensions (150 × 150 × 150 mm and 100 × 100 100 mm). To estimate the characteristics of BPC, an ML model considered a wide range of input features. To build up a predictive model for the slump, six input variables, which include gravel, sand, silty clay, cement, bentonite, and water, were retrieved from the literature. In addition, for modeling compressive and elastic modulus, curing time was added to these six influential input variables.

**Figure 3:**
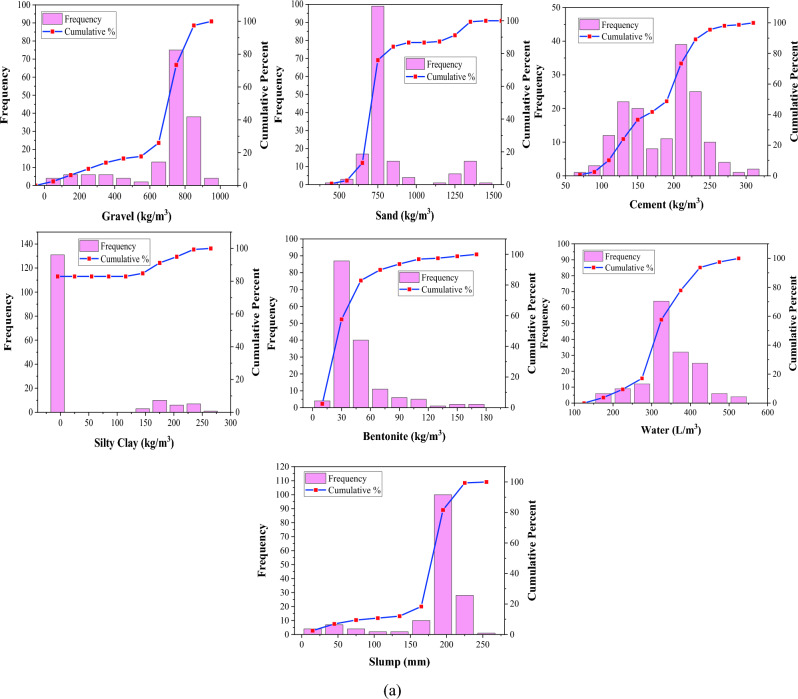

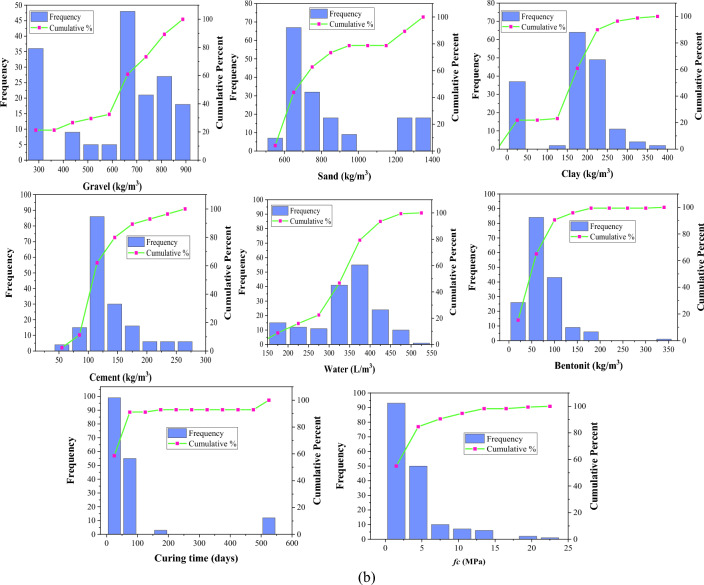

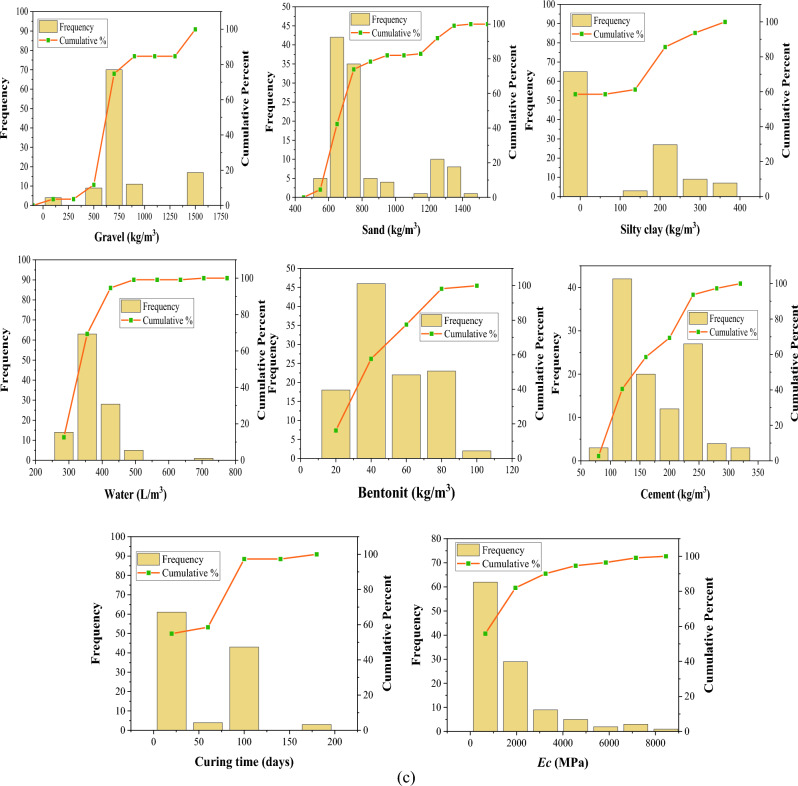
Frequency histograms of variables: (**a**) Slump (**b**) *fc* (**c**) *Ec.*

The distribution of input variables influences the generated model's generalization capabilities. Frequency histograms are provided in Fig. 3 to visualize variable distribution. Tables 1, 2 and 3 summarize the various statistics for the collected datasets of slump, compressive strength, and elastic modulus. The dataset is split into three categories: testing (15%), training (70%), and validation (15%). This data partitioning approach facilitates evaluating the model's performance on new, unseen data, offering a more precise gauge of its real-world applicability. By doing so, it mitigates the risk of overfitting, preventing the model from depending excessively on particular training data patterns. Additionally, it supports model refinement and hyperparameter optimization by furnishing a distinct validation set for comparing and selecting the most effective model configurations^47,52,84^. Each subset of the dataset has comparable statistical characteristics such as standard deviation, variance, mean, and range. These statistical analyses prove that the proposed ML models are usable for a diverse set of data, which broadens their generalization. It is noticeable that only a few research have determined slump, *fc*, and *Ec* for a specific mix proportion. Due to this reason, separate databases have been collected for these three characteristics and are considered for their respective model development.

**Table 1:** Statistical analysis of the slump dataset.

Statistics	Gravel (Kg.m^−3^)	Sand (Kg.m^−3^)	Silty clay (Kg.m^−3^)	Cement (Kg.m^−3^)	Bentonite (Kg.m^−3^)	Water (L.m^−3^)	Slump (mm)
Training (70%)							
Mean	684.958	815.427	34.587	181.780	48.202	349.685	181.688
Standard deviation	206.970	216.642	76.452	49.460	30.057	68.354	45.501
Sample variance	42,836.767	46,933.895	5844.967	2446.340	903.395	4672.248	2070.328
Range	912.000	864.000	260.000	228.000	152.000	347.900	220.000
Testing (15%)							
Mean	728.938	809.063	39.792	172.333	38.188	326.925	186.208
Standard deviation	208.559	172.442	80.305	43.646	15.251	56.345	33.127
Sample variance	43,496.789	29,736.137	6448.868	1905.014	232.586	3174.749	1097.389
Range	926.000	747.000	225.000	150.000	57.000	259.200	179.000
Validation (15%)							
Mean	676.160	793.440	23.400	186.880	37.676	329.723	161.040
Standard deviation	235.618	197.696	65.108	59.313	18.076	77.378	69.056
Sample variance	55,515.973	39,083.673	4239.000	3518.027	326.729	5987.366	4768.790
Range	889.000	928.000	225.000	189.000	85.000	340.000	217.000

**Table 2:** Statistical analysis of compressive strength dataset.

Statistics	Gravel (Kg.m^−3^)	Sand (Kg.m^−3^)	Silty clay (Kg.m^−3^)	Cement (Kg.m^−3^)	Bentonite (Kg.m^−3^)	Water (L.m^−3^)	Curing time (days)	*fc* (MPa)
Training (70%)								
Mean	615.3342	835.8254	158.3772	130.7456	75.74561	337.3454	86.31579	3.502456
Standard deviation	187.2431	250.8098	94.75048	36.82506	41.73342	77.82741	138.8061	2.553283
Sample variance	35,059.99	62,905.58	8977.653	1356.085	1741.678	6057.106	19,267.12	6.519254
Range	580	781	380	202	304	347.9	533	13.74
Testing (15%)								
Mean	679.9621	778.2034	154.8276	137.8966	66.41379	326.7959	87.72414	5.685172
Standard deviation	168.8335	229.3582	102.4389	46.04217	31.06849	88.56716	129.942	5.528141
Sample variance	28,504.76	52,605.17	10,493.72	2119.882	965.2512	7844.141	16,884.92	30.56034
Range	580	781	280	202	122	297.9	533	20.19
Validation (15%)								
Mean	654.4222	917.4593	174.2593	146.4444	64.77778	344.1211	67.59259	4.103704
Standard deviation	192.6646	289.7691	70.96456	42.65184	30.45089	60.193	99.91968	4.410454
Sample variance	35,226.23	83,966.14	5035.969	1819.179	927.2564	3623.198	9983.943	19.45211
Range	580	781	310	180	124	277.85	533	20.77

**Table 3:** Statistical analysis of the elastic modulus dataset.

Statistics	Gravel (Kg.m^−3^)	Sand (Kg.m^−3^)	Silty clay (Kg.m^−3^)	Cement (Kg.m^−3^)	Bentonite (Kg.m^−3^)	Water (L.m^−3^)	Curing time (days)	*Ec* (MPa)
Training (70%)								
Mean	785.588	798.274	99.855	165.928	50.245	367.767	61.957	1825.915
Standard deviation	310.351	235.661	119.539	51.926	19.322	60.462	40.335	1749.309
Sample variance	96,317.836	55,536.138	14,289.685	2696.333	373.356	3655.696	1626.925	3,060,080.52
Range	1519.600	848.000	370.000	250.000	80.000	430.000	173.000	7686.720
Testing (15%)								
Mean	930.290	900.859	72.381	197.905	37.181	356.110	43.095	1484.000
Standard deviation	420.056	318.772	137.510	67.107	17.867	54.500	27.630	1016.307
Sample variance	176,447.408	101,615.448	18,909.048	4503.290	319.234	2970.290	763.390	1,032,880.43
Range	1547.700	990.000	380.000	200.000	85.200	205.000	83.000	3896.480
Validation (15%)								
Mean	735.862	768.878	99.048	169.476	44.105	367.462	48.667	1718.613
Standard deviation	315.819	217.178	113.398	61.712	19.018	60.572	29.949	1378.513
Sample variance	99,741.440	47,166.438	12,859.048	3808.362	361.681	3668.990	896.933	1,900,297.05
Range	1519.600	827.000	330.000	200.000	64.000	220.000	62.000	4673.750

### MEP model development

**Figure 4:**
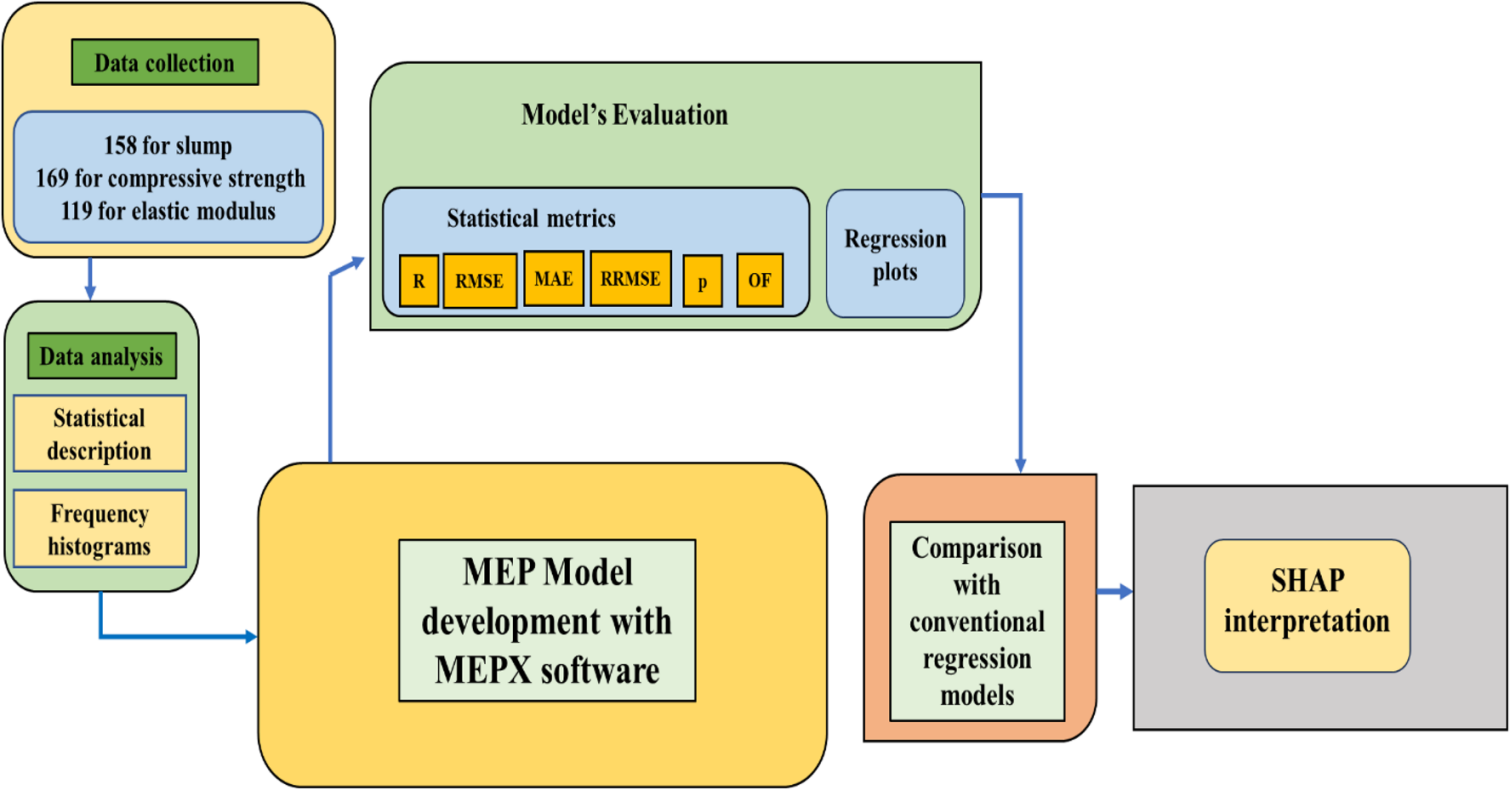
Flowchart of the methodology followed in the present study.

The methodology used in this research is outlined in Fig. 4. Several MEP setting variables must be defined prior to building a valid and adaptive model. The setting variables are chosen by prior recommendations and a trial-and-error procedure^85^. The number of developed programs is determined by the population size. A large-scale population model can be more complex, but it is more exact and reliable, and it takes longer to reach convergence. However, if the size increases above a certain range, the model may overfit. Table 4 shows the setup variables that were used for the model constructed in this work. The function just comprises the simple mathematical operators (ln, exp, -, × , ÷ , +) for simplicity in the final formulations. The number of generations indicates the accuracy of the method before it is discontinued. The model for simulation with the fewest errors will be produced by a multi-generation run. Various variable combinations were used to optimize the model, and the optimum combination was chosen to offer an outcome model with the lowest errors, as shown in Table 4. The main challenge with ML prediction simulation is the over-fitting of the prediction model. Whenever utilized with original data, the model performs well; however, when given unknown data, the model performs significantly worse. To avoid overfitting, it has been suggested that the model be evaluated using previously unknown data^85,86^. As a result, the data is proportionately divided into three groups. Following validation, the model is evaluated on the dataset that was not used in the training of the model. The database was divided into three subsets, i.e., 15% for testing, 15% for validation, and 70% for training. The generated models perform excellently across all datasets. In the current study, the MEPX tool (version. 2023.3.5) was used to carry out MEP modeling.

**Table 4:** Set up of parameters for MEP algorithm.

Parameter	Set up
Sub-population size	250
Mutation probability	0.01
Number of generations	1000
Operators	× , ÷ , + , − , ln, exp
Number of sub-populations	50
Tournament size	40
Variable	0.5
Length of code	0.5
Probability of crossover	0.9
Fitness function	MAE

Initially, the modeling process generates optimal solutions for the population. The procedure is repeated, with every iteration getting closer to a solution. The fitness of each successive generation is determined. The MEP modeling process carries on until the fitness value does not change. If the outcomes are not precise, the operation is iterated by progressively increasing the size of the population and tuning other hyperparameters. After evaluating the fitness function of every model, the model with the lowest fitness is chosen. It should be noted that the evolution time and the number of generations have a considerable impact on the accuracy of the suggested model. Due to the addition of new features to the framework, a model will be iterated indefinitely using these approaches. However, in the current study, the model has completed either the change in function was less than 0.1% or after 1000 generations. The hyperparameters setup of the suggested MEP model is provided in Table 4.

### Model performance assessment

The models' effectiveness is assessed by calculating numerous statistical error metrics. Multiple performance metrics such as R, RMSE, MAE, RRMSE, RSE, and performance index (ρ) are used to check the accuracy of the MEP model, as given in Eqs. (1–6). Another approach to prevent model overfitting is to choose the optimum model by reducing the objective function (OF)^88^,^89^.

1 $${\text{RMSE}} = \sqrt {\frac{{\mathop \sum \nolimits_{{\text{i = 1}}}^{{\text{n}}} \left( {{\text{ei}} - {\text{mi}}} \right)^{{2}} }}{{\text{n}}}}$$

2 $${\text{MAE}} = \frac{{\mathop \sum \nolimits_{{{\text{i}} = {1}}}^{{\text{n}}} \left| {{\text{ei}} - {\text{mi}}} \right|}}{{\text{n}}}$$

3 $${\text{R}} = \frac{{\mathop \sum \nolimits_{{{\text{i}} = 1}}^{{\text{n}}} \left( {{\text{ei}} - {\bar{\text{e}i}}} \right)\left( {\text{mi}} - ~{\bar{{\text{m}i}}} \right)~}}{{\sqrt {\mathop \sum \nolimits_{{i = 1}}^{n} \left( {{\text{ei}} - ~{\bar{\text{e}i}}} \right)^{2} \mathop \sum \nolimits_{{{\text{i}} = 1}}^{{\text{n}}} \left( {{\text{mi}} - {\bar{\text{m}i}}} \right)^{2} ~} }}$$

4 $${\text{RRMSE}} = \frac{{1}}{{\left| {{\overline{\text{e}}}} \right|}} \cdot \sqrt {\frac{{\mathop \sum \nolimits_{{\text{i = 1}}}^{{\text{n}}} \left( {{\text{ei}} - {\text{mi}}} \right)^{{2}} }}{{\text{n}}}}$$

5 $$\uprho = \frac{{{\text{RRMSE}}}}{{\text{1 + R}}}$$

6 $${\text{OF}} = \left( {\frac{{{\text{n}}_{{\text{T}}} - {\text{n}}_{{\text{v}}} }}{{\text{n}}}} \right){\text{p}}_{{\text{T}}} + 2\left( {\frac{{{\text{n}}_{{\text{v}}} }}{{\text{n}}}} \right){\text{p}}_{{\text{V}}}$$

where ei shows actual data and mi shows model data of actual while n denotes the number of collected values. Whereas ${\bar{\text{{ei}}}}$ and ${\bar{\text{{mi}}}}$ represent the mean of experimental and predicted values, respectively. The training and validation sets are represented by the subscripts T and V, respectively. R measures the correlation between estimated and actual values^87^, and a value greater than 0.8 shows a strong connection between anticipated and actual results^88,89^. However, because R is insensitive to the division or multiplication of data by a constant number, it is insufficient as a check of the overall model efficacy. The RMSE and MAE calculate the mean magnitude of the errors. Each variable, though, has its own significance. A larger RMSE value indicates that the frequency of estimations with substantial errors is significantly greater than expected and should be decreased. On the other hand, MAE provides minimum weight to higher error and is always lower than RMSE.

The MEP model used in this study is also assessed via the OF to determine the overall efficiency because OF takes into account the influence of RMSE, R, and the total number of collected values. The values OF range from 0 to infinity. A model is considered best if $\uprho$ and OF are both 0.2^88^. The OF considers three parameters, namely R, RRMSE, and the proportion of data in validation and training sets. Consequently, the least value signifies a model's greater performance. Furthermore, the MEP model was externally validated using criteria suggested in the literature, as shown in Table 5.

**Table 5:** External validation requirements.

S. no	Expression	Conditions	Suggested by
1	${\text{k = }}\frac{{\sum\limits_{{{\text{i}} = 1}}^{{\text{n}}} {\left( {{\text{ei}} \times {\text{mi}}} \right)} }}{{{\text{e}}_{{\text{i}}}^{2} }}$	$\text{0.85 < k < 1.15}$	^93^
2	${\text{k}}' = \frac{{\mathop \sum \nolimits_{{{\text{i}} = 1}}^{{\text{n}}} \left( {{\text{ei}}~ \times {\text{mi}}} \right)}}{{{\text{mi}}^{2} }}$	$0\text{.85 < k < 1.15}$	^93^
3	${\text{R}}_{{\text{m}}} = {\text{R}}^{2} \times \left( {1 - \sqrt {\left| {{\text{R}}^{{2~}} - {\text{R}}_{{\text{o}}}^{2} } \right|} } \right)$ where ${\text{R}}^{2} = 1 - ~\frac{{\mathop \sum \nolimits_{{{\text{i}} = 1}}^{{\text{n}}} \left( {{\text{mi}} - ~{\text{e}}_{{\text{i}}}^{^\circ } } \right)^{2} }}{{\mathop \sum \nolimits_{{{\text{i}} = 1}}^{{\text{n}}} \left( {{\text{mi}} - ~{\text{m}}_{{\text{i}}}^{{\text{o}}} } \right)^{2} }}$ ${\text{e}}_{{\text{i}}}^{^\circ } = {\text{k}} \times ~{\text{m}}_{{\text{i}}}$	${\text{R}}_{\text{m}}\text{>0.5}$ ${\text{R}}^{{2}} \cong 1$	^94^
	${\text{R}}_{^\circ }^{2} = 1 - ~\frac{{\mathop \sum \nolimits_{{{\text{i}} = 1}}^{{\text{n}}} \left( {{\text{ei}} - {\text{m}}_{{\text{i}}}^{^\circ } } \right)^{2} }}{{\mathop \sum \nolimits_{{{\text{i}} = 1}}^{{\text{n}}} \left( {{\text{ei}} - {\text{~e}}_{{\text{i}}}^{{\text{o}}} } \right)^{2} }}$ ${\text{m}}_{{\text{i}}}^{^\circ } = {\text{k'}} \times ~{\text{e}}_{{\text{i}}}$	${\text{R}}_{^\circ }^{2} \cong 1$	

## Results and discussion

The MEP algorithm was employed to construct predictive models for various properties of bentonite plastic concrete. These models were meticulously developed with a hyperparameter configuration comprising a sub-population size of 250, generations of 1000, a mutation probability of 0.9, and sub-populations of 50. Additionally, mathematical operators including + , −, /, Inv, and exp were utilized in the model construction process. The optimized MEP code for future prediction of slump, *fc*, and *Ec* has been compiled and is conveniently accessible in the supplementary materials under Tables S4–S6. These codes provide a comprehensive overview of the generated code, facilitating accurate and efficient forecasting of slump, *fc*, and *Ec*.

### Outcomes of MEP modeling

**Figure 5:**
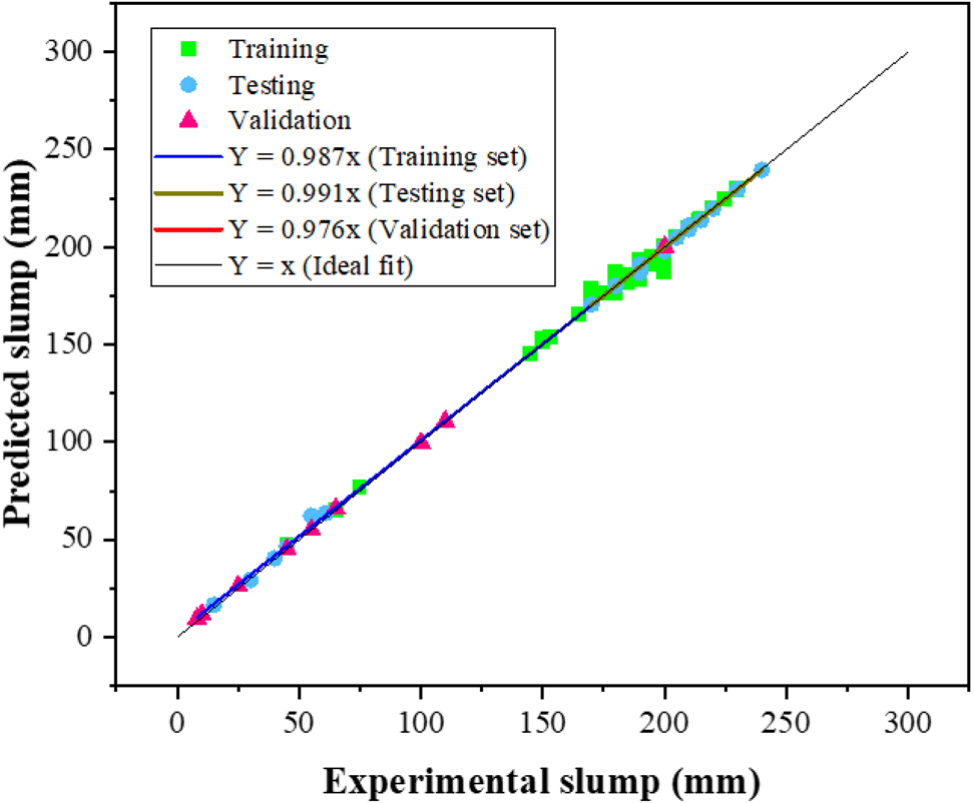
MEP prediction model comparison with experimental data of slump.

Figure 5 displays the comparison model forecasted and experimental values of the slump. The plot also includes the expressions for regression lines. In perfect condition, the regression slope should be approached close to 1. Figure 5 illustrates a significant correlation between original and modeled values, as evidenced by slopes of 0.987, 0.991, and 0.976 for the training, validation, and testing phases, respectively. Moreover, the values are relatively similar and near to perfect matching, showing that the MEP model is trained effectively and has a better prediction performance, i.e., it works similarly very well with new data.

**Figure 6:**
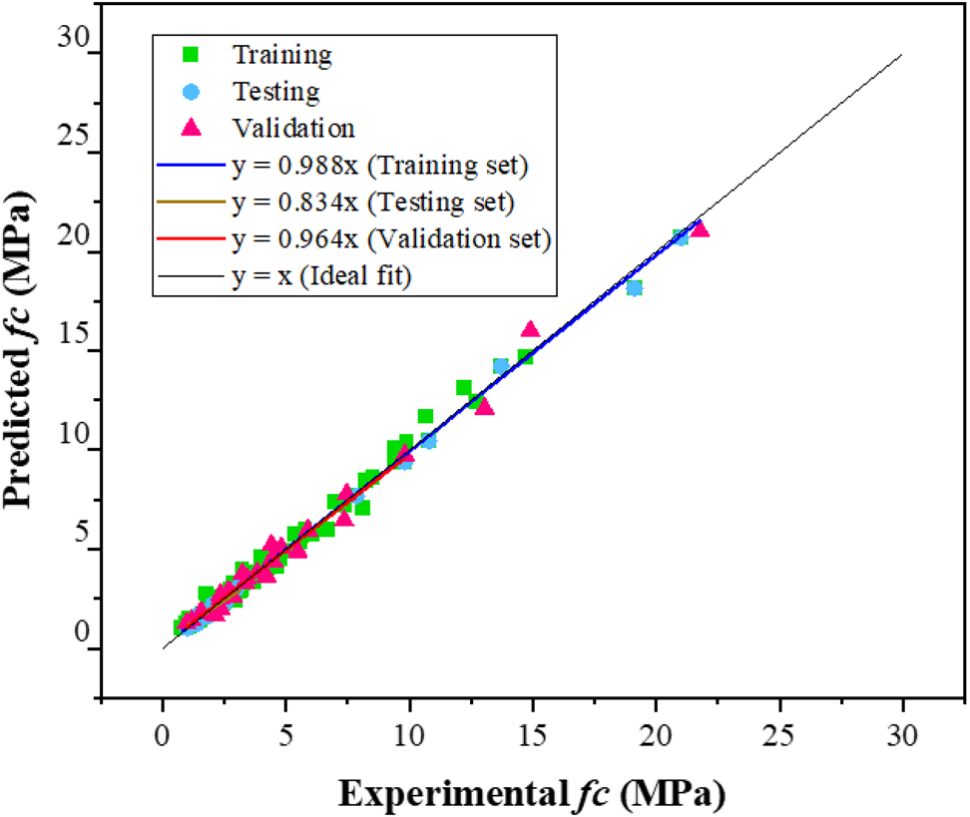
MEP prediction model comparison with experimental data of *fc.*

The *fc* findings have also been compared to experimental values of *fc*, as shown in Fig. 6. The resulting model appears to have undergone effective training on the input data, as evidenced by its ability to generate precise predictions for the actual *fc*. All three sets of data have almost optimal regression line slopes (0.988, 0.834, and 0.984). This model, like the one for the slump, does very well on test data. This demonstrates that the concern of the model being overfitted has been much reduced. The greater the number of data points, the more accurate and generalizable the outcomes will be^90^. The largest number of points possible (169) were chosen for *fc* in the compiled database, resulting in a high level of precision with the least statistical errors.

**Figure 7:**
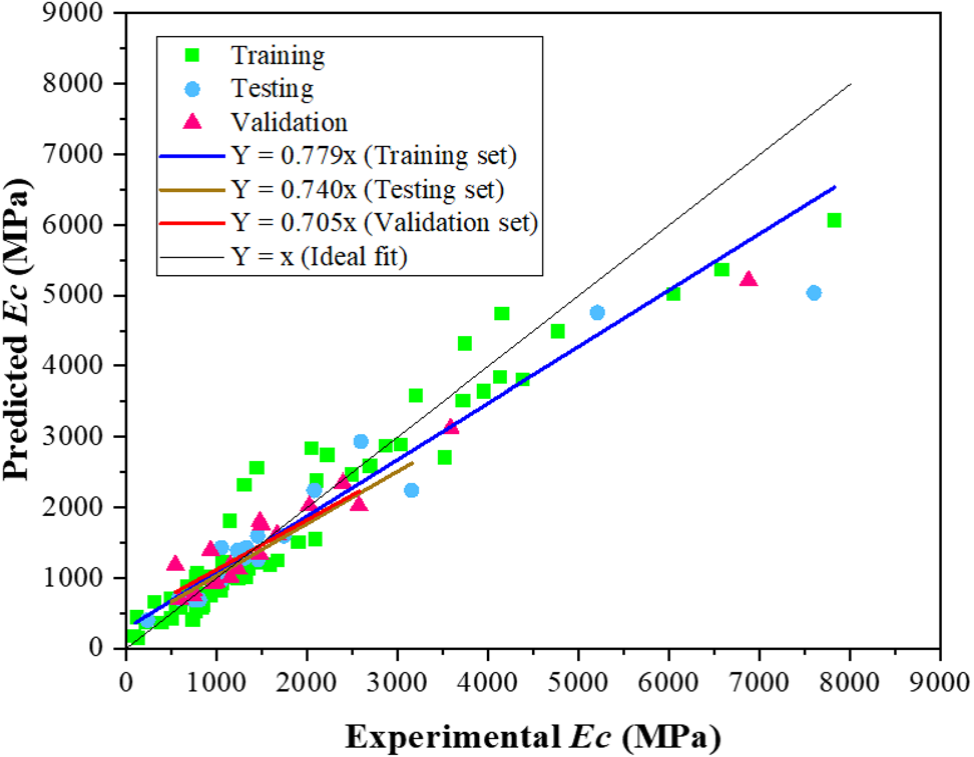
MEP prediction model comparison with experimental data of *Ec.*

Similarly, Fig. 7 provides a comparison of the model and experimental results of *Ec*. In contrast, to slump and *fc* models, the MEP model for *Ec* exhibited a comparatively lower regression slope as shown in Fig. 7. According to Gholampour et al.^90^, the precision and efficacy of the model are heavily influenced by the number of dataset points. In the current work, a greater number of datasets (111) were obtained from the available published work and used for the suggested model, resulting in improved accuracy.

### Performance evaluation of MEP models

**Figure 8:**
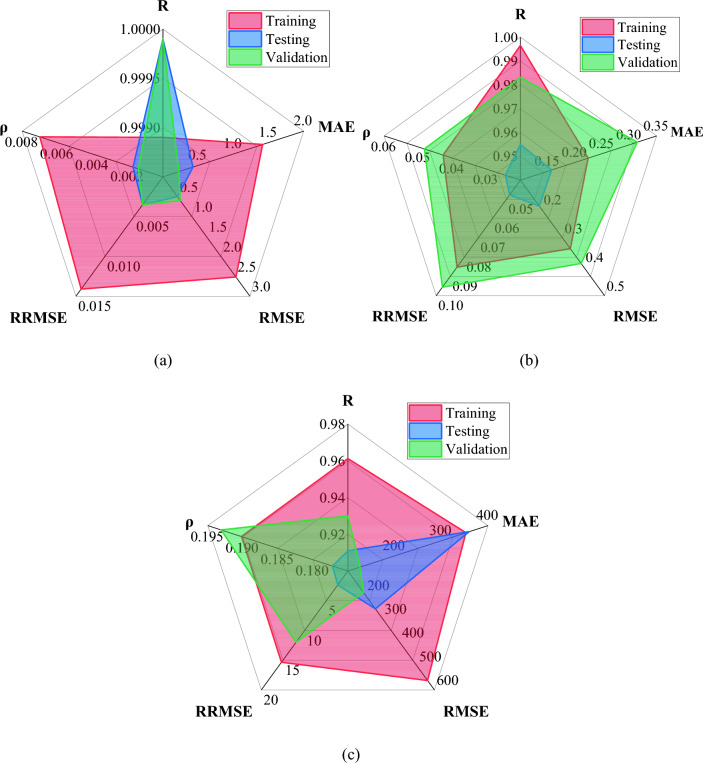
Radar plots presenting the performance of MEP models: (**a**) Slump, (**b**) *fc*, (**c**) *Ec.*

The number of data points required to construct a model is crucial because it affects the model's validity. The data set proportion to the number of inputs should be 3 for a satisfactory model, and a ratio of 5 is preferred^90,91^. In this study, the ratios are 26.3, 24.1, and 15.9 for the slump, *fc*, and *Ec*, respectively. As discussed previously, the performance of all three models is assessed by using various statistical measures (R, MAE, RMSE, RSE, RRMSE, ρ, and OF)*.* The values of all these error measurements for the three models are provided in Table 6 and illustrated in Fig. 8. The table provides a good correlation between the model estimated and actual values, as R-values are closer to 1 (ideal condition) for the three suggested models. The MAE, RSE, and RMSE values for the three datasets are notably lower, which indicates the good precision and generalization ability of MEP models.

**Table 6:** Various statistical calculations of the MEP model.

Model	Subset	R	MAE	RMSE	RRMSE	ρ	OF
Slump	Training	0.9989	1.4175	2.5176	0.0141	0.0070	0.0038
	Testing	0.9999	0.4272	0.5018	0.0035	0.0017	
	Validation	0.9999	0.2262	0.6007	0.0035	0.0014	
*fc*	Training	0.9965	0.2245	0.3374	0.0852	0.0427	0.0471
	Testing	0.9550	0.1574	0.1897	0.0477	0.0244	
	Validation	0.9831	0.3146	0.3886	0.0956	0.0482	
*Ec*	Training	0.9612	352.9337	560.2367	15.3485	0.1902	0.1662
	Testing	0.9110	359.6862	258.9647	2.3486	0.1772	
	Validation	0.9300	126.0827	189.6300	11.9749	0.1931

It is shown that the values of RRMSE in the three sets of slump models are lower than 0.2, indicating that the slump model is in the excellent range. The values of $\uprho$ are less than 0.20 for all sets of slump and compressive strength models, demonstrating that the MEP models are accurate and suitable for predicting the output. However, these values of $\uprho$ are a little high for the elastic modulus model. OF for the slump, *fc,* and *Ec* models are 0.0453, 0.0471, and 0.1662, respectively. These values are quite close to 0, substantiating the accuracy and indicating that the issue of overfitting for the models has been adequately handled.

**Figure 9:**
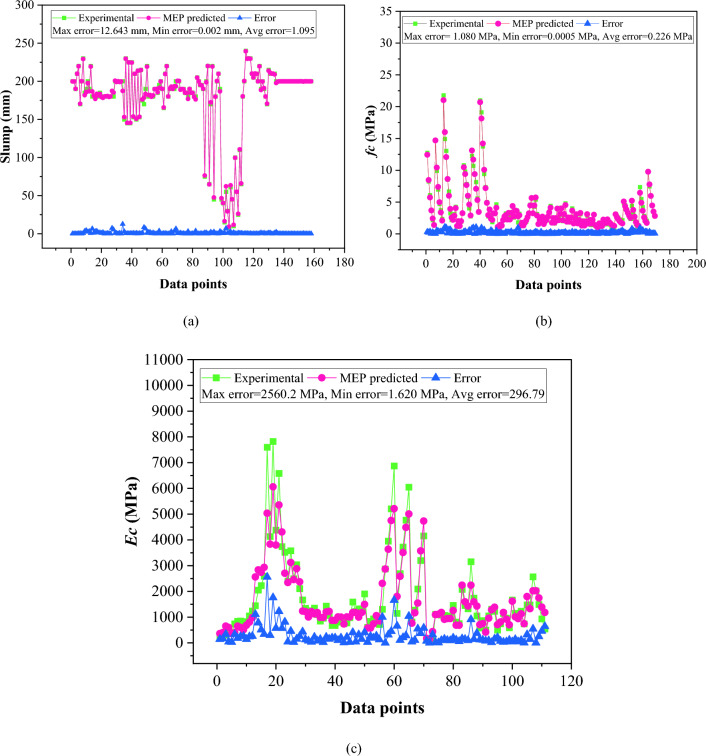
Representation of error in the established models: (**a**) slump; (**b**) *fc*; (**c**) *Ec.*

Figure 9 shows the absolute error in each MEP model to explain the statistics of absolute errors. The mean absolute error values for the slump, *fc,* and *Ec* are 1.095 mm, 0.226 MPa, and 296.79 MPa, respectively, with a maximum error of 12.64 mm, 1.08 MPa, and 2560.2 MPa. It is worth noting that the occurrence of maximum error is very low. In addition, the predicted values of MEP models closely followed the trend of the experimental values.

### External validation of MEP model

Table 7 represents the numbers of the additional criteria used for model validations. It has been proposed that the slopes of regression lines should be close to 1^92^. Roy and Roy^93^ proposed another criterion of Rm to measure the external reliability of the model. When the value of Rm is higher than 0.5, this criterion is satisfied. Table 7 illustrates that the MEP model meets the additional validation criteria, showing that the MEP algorithm is accurate and has better predictive potential. Thus, the formulated MEP models have the potential to accurately and precisely predict the workability and strength properties of BPC.

**Table 7:** Various external validation values of the proposed models.

S.No	Parameter	Slump	*fc*	*Ec*
(1)	*k*	0.99	1.04	1.12
(2)	*k’*	1.10	0.95	0.87
(3)	*R*^*2*^	0.99	0.97	0.94
(4)	*R*_*0*_^*2*^	0.99	0.99	0.96
(5)	*Rm*	0.98	0.80	0.75

### Comparing the MEP model with statistical regression models

In this study, non-linear (NLR) and linear regression (LR) models were constructed using similar databases to predict the characteristics of BPC. The outcomes were compared with MEP models. The RMSE and ρ values are lower for the MEP model compared to the regression models for all three datasets.

**Figure 10:**
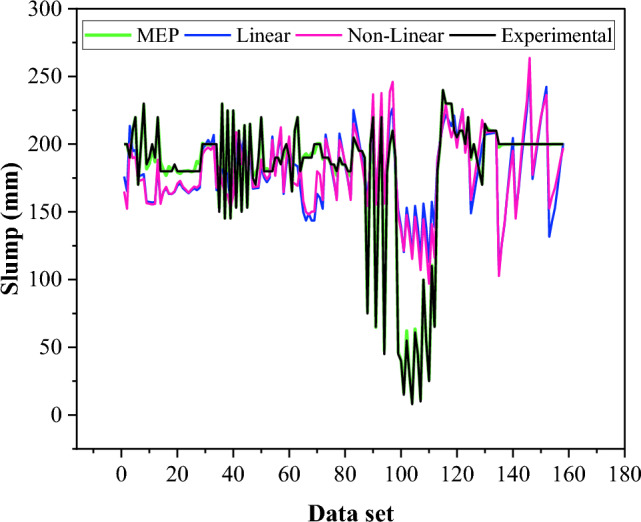
Comparison of slump predicted by MEP with LR and NLR models.

The formulations to estimate the slump of BPC using LR and NLR analysis are provided in Eqs. (7–8). The results of NLR and LR regression analysis are compared with the MEP model for the slump and shown in Fig. 10. The RMSE_training_ of the MEP model for the slump is 95.2% lower than that of the linear regression, which shows the accuracy and reliability of the MEP model. It is worth noticing from Fig. 10 that the regression model failed to capture the lower value of the slump.

7 $$\begin{aligned} \left( {{\text{Slump}}} \right)_{{{\text{LR}}}} & = {1}0{6}.{7 } + \, 0.0{38 }\left( {{\text{gravel}}} \right) + \, 0.00{5 }\left( {{\text{sand}}} \right) \, + \, 0.0{51}\left( {\text{silty clay}} \right) \, \\ & \quad - \, 0.{37 }\left( {{\text{cement}}} \right) \, + 0.0{86 }\left( {{\text{Bentonite}}} \right) \, + \, 0.{3}0 \, \left( {{\text{water}}} \right) \\ \end{aligned}$$

8 $$\begin{aligned} \left( {{\text{Slump}}} \right)_{{{\text{NLR}}}} & = { 81}.{8 } + { 1} \times {1}0^{{ - {6}}} \left( {{\text{gravel}}} \right)^{{{2}.{6}}} + { 3}.{2} \times {1}0^{{ - {3}}} \left( {{\text{sand}}} \right)^{{{1}.{23}}} + \, 0.{17 }\left( {\text{silty clay}} \right)^{{0.{94}}} \\ & \quad - {6} \times {1}0^{{ - {5}}} \left( {{\text{cement}}} \right)^{{{2}.{5}}} + \, 0.{28 }\left( {{\text{bentonite}}} \right)^{{0.{75}}} + {1} \times {1}0^{{ - {3}}} \left( {{\text{water}}} \right)^{{{1}.{89}}} \\ \end{aligned}$$

**Figure 11:**
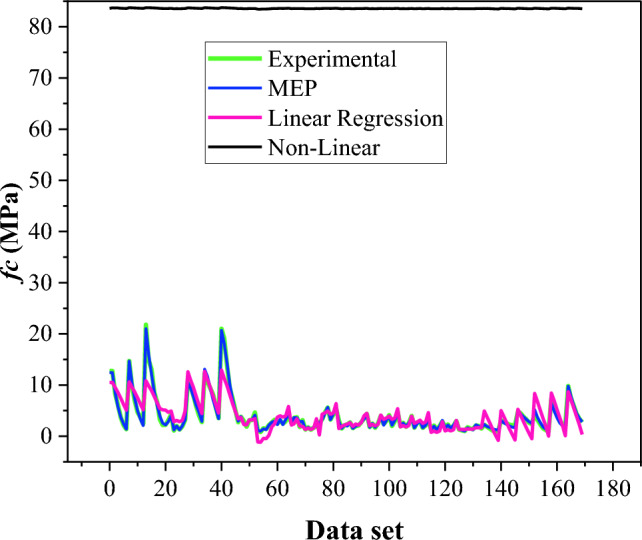
Comparison of *fc* predicted by MEP with LR and NLR models.

Similarly, based on the same dataset, LR and NLR analyses are conducted for the compressive strength of BPC. LR and NLR formulations for *fc* of BPC are shown as Eqs. (9–10). MEP-predicted values for compressive strength are compared with LR and NLR, as provided in Fig. 11. The statistical errors for the MEP model of *fc* are considerably lower than those of regression models. The RMSE_traning_ of the MEP model is 52% less than that of the regression model, which indicates the inaccuracy of the conventional regression model. It is worth noticing that the non-linear regression model for *fc* produced similar values of outcomes throughout all the dataset data, as represented by the nearly straight line. This provides the inaccuracy of the NLR model to forecast the *fc* of BPC.

9 $$\begin{aligned} \left( {fc} \right)_{LR} & = \, - {14}.{9 } + { 1}.{4} \times {1}0^{{ - {2}}} \left( {{\text{gravel}}} \right) \, + { 7}.{1} \times {1}0^{{ - {3}}} \left( {{\text{sand}}} \right) \, \\ & \quad + { 1}.{3} \times {1}0^{{ - {3}}} \left( {\text{silty clay}} \right) \, + { 5}.{1} \times {1}0^{{ - {2}}} \left( {{\text{cement}}} \right) \, \\ & \quad + { 3} \times {1}0^{{ - {4}}} \left( {{\text{bentonite}}} \right) \, {-}{ 9}.{6} \times {1}0^{{ - {3}}} \left( {{\text{water}}} \right) \, + { 4}.{1} \times {1}0^{{ - {3}}} \left( {\text{curing time}} \right) \\ \end{aligned}$$

10 $$\begin{aligned} \left( {fc} \right)_{NLR} & = { 82}.{3 } + { 8}.{7} \times {1}0^{{ - {2}}} \left( {{\text{gravel}}} \right)^{{0.{28}}} + { 4}.{1} \times {1}0^{{ - {2}}} \left( {{\text{sand}}} \right)^{{0.{35}}} \\ & \quad + { 1}.{24} \times {1}0^{{ - {8}}} \left( {\text{silty clay}} \right)^{{{2}.{65}}} + \, 0.{12 }\left( {{\text{cement}}} \right)^{{0.{27}}} + { 9}.{1} \times {1}0^{{ - {8}}} \left( {{\text{bentonite}}} \right)^{{{2}.{3}}} \\ & \quad {-}{ 7}.{9} \times {1}0^{{ - {2}}} \left( {{\text{water}}} \right)^{{0.{25}}} + { 8}.{1} \times {1}0^{{ - {2}}} \left( {\text{curing time}} \right)^{{0.{12}}} \\ \end{aligned}$$

**Figure 12:**
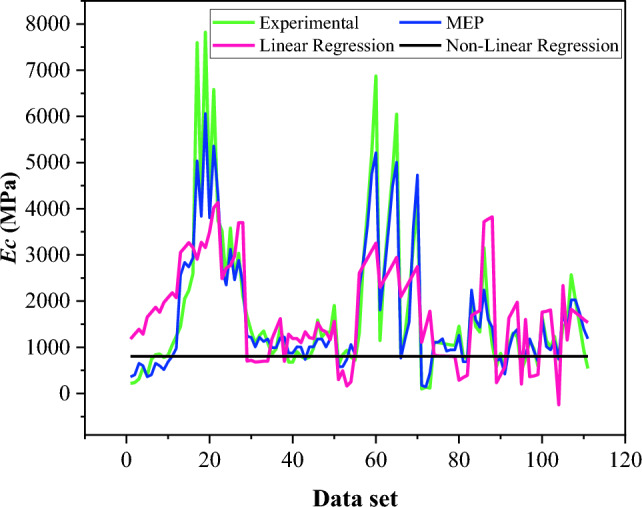
Comparison of *Ec* predicted by MEP with LR and NLR models.

The formulations of LR and NLR for the elastic modulus of BPC are given as Eqs. (11–12). The outcomes of elastic modulus regression analysis are compared with the MEP model and experimental data, as depicted in Fig. 12. The RMSE_training_ of the MEP model for *Ec* is 60% lower than that of linear regression, which shows the excellent capability of MEP model to precisely forecast the elastic modulus of BPC.

11 $$\begin{aligned} (Ec)_{{{\text{LR}}}} & = { 896}.{1 } + { 22}.{6 }\left( {{\text{gravel}}} \right) \, + \, 0.{84 }\left( {{\text{sand}}} \right){-}{ 21}.{1 }\left( {\text{silty clay}} \right) \, \\ & \quad + { 5}.{12}\left( {{\text{cement}}} \right){-} \, 0.{6}\left( {{\text{bentonite}}} \right) \, + \, 0.{3 }\left( {{\text{water}}} \right) \, {-}{ 1}.{1}\left( {\text{curing time}} \right) \\ \end{aligned}$$

12 $$\begin{aligned} \left( {Ec} \right)_{NLR} & = { 762}.{3 } + { 12}.{6 9}\left( {{\text{gravel}}} \right)^{{0.{13}}} {-} \, 0.{4}\left( {{\text{sand}}} \right)^{{ - {1}.{86}}} - { 6} \times {1}0^{{ - {2}}} \left( {\text{silty clay}} \right)^{{0.{39}}} + { 26 }\left( {{\text{cement}}} \right)^{{0.00{7}}} \\ & \quad + { 4}.{48} \times {1}0^{{ - {6}}} \left( {{\text{bentonite}}} \right)^{{{1}.{4}}} & \quad + \, 0.{5}\left( {{\text{water}}} \right)^{{0.0{9}}} {-}{ 8}.{7} \times {1}0^{{ - {8}}} \left( {\text{curing time}} \right)^{{{2}.{1}}} \\ \end{aligned}$$

These findings imply that MEP-based models outperform both LR and NLR models. The reason for this is that these statistical regression procedures have limits, such as the actual issue being linked to a forecast model by certain pre-defined functions. In contrast, the outcomes of MEP-based modeling, demonstrate that the models have a great generalization capability and, most importantly, less error than the regression models. Hence, these limitations impede the utilization of statistical regression models for predictive tasks.

### Comparison of the developed models with literature models

To date, several soft-computing models have been developed to predict the properties of bentonite plastic concrete. The majority of developed models have primarily focused on predicting the *fc* of BPC. However, it is noteworthy that despite the significance of slump, most studies have not delved into developing prediction models for this parameter, with Amlashi et al.^83^ being an exception. To facilitate a precise comparison between existing models from the literature and the established models in this study, two statistical metrics (R, RMSE) were selected, as given in Table 8.

**Table 8:** Comparison of the established models and existing literature models.

Author(s)	Method	Investigated properties	Dataset	Feature importance method	Performance	
					R	RMSE
Ghanizadeh et al.^14^	ANN	*fc*	144	Sensitivity and parametric analyses	0.995	0.4510
	SVM				0.992	0.6907
Amlashi et al.^65^	SVM	*fc*	169	Sensitivity analysis	0.992	0.461
	GMDH				0.927	1.120
	MGGP				0.973	0.863
	RSM				0.953	1.121
Amlashi et al.^94^	ANN	*fc*	387	Cosine Amplitude Method	0.961	0.500
	ANN-PSO				0.978	0.353
	SVM				0.946	0.723
	SVM-PSO				0.942	0.469
	ANFIS				0.925	0.845
	ANFIS-PSO				0.954	0.572
Amlashi et al.^83^	ANN	Slump	158	Cosine amplitude method	0.966	11.680
	MARS				0.872	16.646
	M5Tree				0.926	18.030
	ANN	*fc*	169		0.986	0.574
	MARS				0.982	0.656
	M5Tree				0.951	0.963
	ANN	*Ec*	119		0.958	472.252
	MARS				0.793	997.634
	M5Tree				0.896	765.052
Present study	MEP	Slump	158	SHAP method	0.9999	0.5018
	MEP	*fc*	169		0.9550	0.1897
	MEP	*Ec*	119		0.9110	258.9647

As given in Table 8, a comparative analysis between the highest-performing model for predicting slump models in this study (i.e., MEP model) and the top-performing model from the literature (i.e., ANN model by Amlashi et al.^83^). The RMSE value of the MEP model was reduced by 95.70% compared to the top-performing model (ANN model developed by Amlashi et al.^83^) in the literature for slump prediction of BPC. Similarly, the RMSE value of the MEP model for compressive strength is 46.26% lower than that of the best prediction model in the literature (ANN-PSO developed by Amlashi et al.^94^). Furthermore, the reduction in RMSE for *Ec* is 45.16% in the MEP model developed in the present study compared to the most accurate model found in the literature (ANN model developed by Amlashi et al.^83^). The developed MEP model exhibited superior accuracy in predicting both the workability and strength properties of bentonite plastic concrete. The MEP model's performance surpassed that of all models reported in the literature, demonstrating its efficacy in optimizing predictions for bentonite plastic concrete. This accuracy signifies a significant advancement in predictive modeling of BPC, promising enhanced reliability for engineering applications.

Moreover, while the bulk of the research concentrated on constructing ML models for predicting BPC properties, it overlooked the crucial aspect of model interpretation. The transparency of ML models is pivotal for engendering trust among end-users. Although several literature studies conducted sensitivity analyses to gauge the importance of individual features in predicting BPC properties, these analyses primarily provide feature significance and do not delve into the internal mechanisms of the models or the complex interrelationships among these features. Hence, this study employed SHAP analysis to interpret the forecasts of the developed models, thereby augmenting their transparency. Overall, this study not only provides models with superior accuracy compared to existing literature models but also enhances model interpretability.

### SHAP interpretability of the models

Lundberg and Lee^95^ developed an approach for analyzing ML models that utilize the concept of Shapely Additive explanations (SHAP). The SHAP-based approach was established to determine each feature's proportionate relevance to the output and to determine if the feature enhances the output favorably or unfavorably^96,97^. References^96,97^ give a thorough explanation of the SHAP method. The SHAP value shows how much each input feature contributed to the results. This approach is equivalent to parametric analysis, in which a particular parameter is changed while others are kept constant to assess how modifications to one input variable are impacting the result.

**Figure 13:**
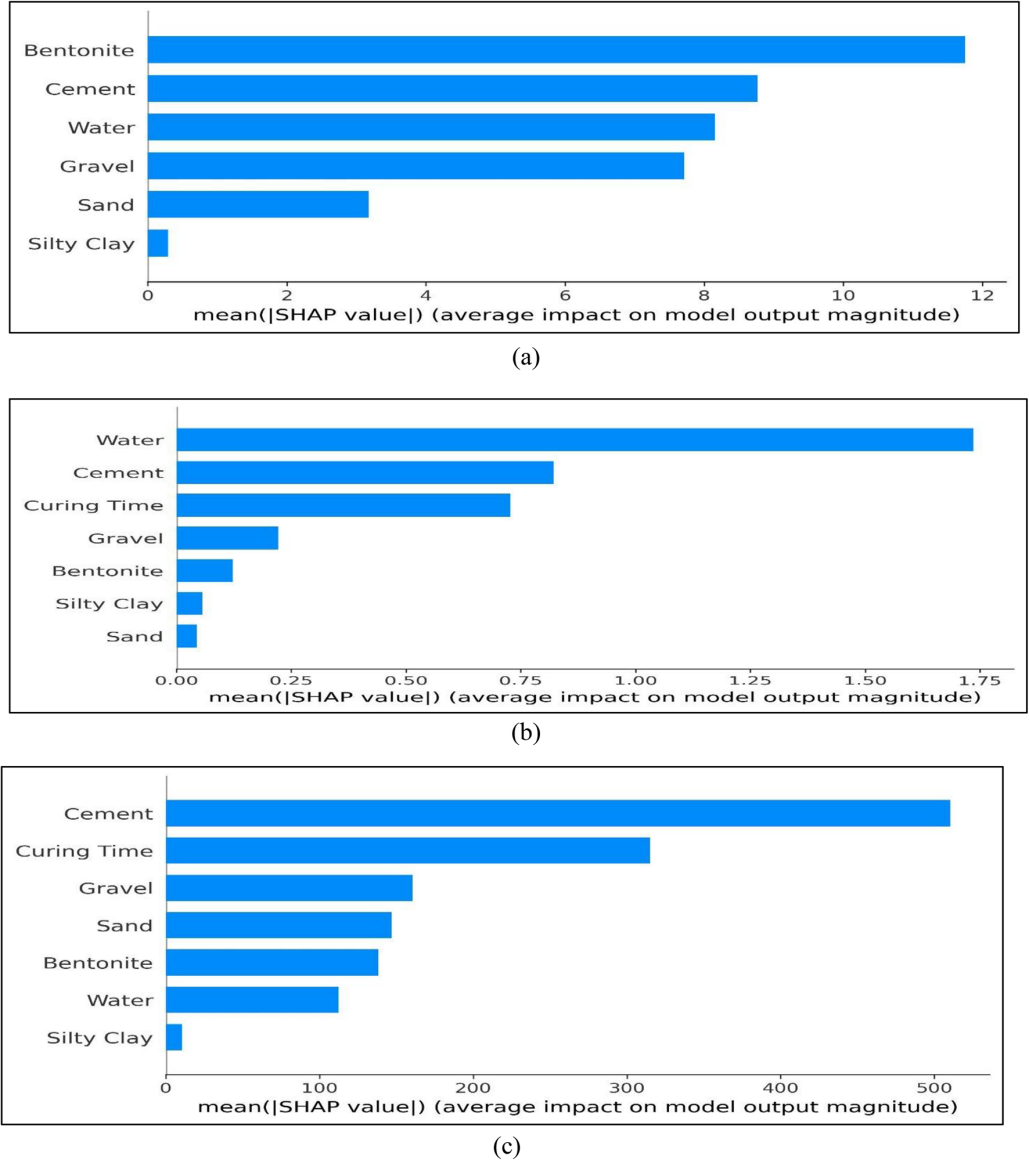
Importance of various input variables: (**a**) Slump; (**b**) *fc*; (**c**) *Ec.*

**Figure 14:**
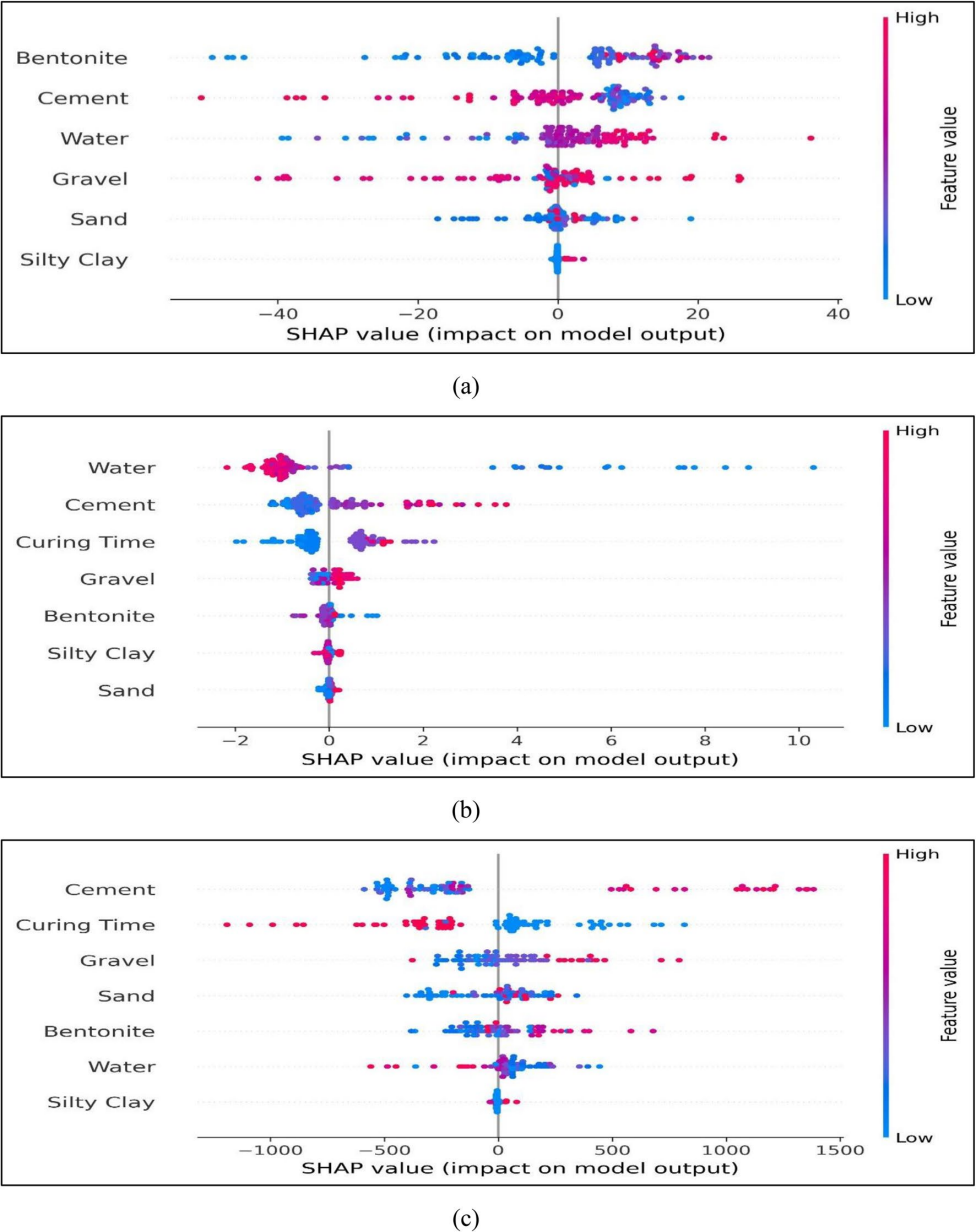
SHAP values of input variables: (**a**) slump; (**b**) *fc*; (**c**) *Ec.*

The mean SHAP values provided in Fig. 13 show the importance of the input parameter. As illustrated in Fig. 13a, bentonite has a relatively greater contribution in output (slump) followed by the rest of the input variables. Similarly, water has relatively more contribution than other input variables in compressive strength, as illustrated in Fig. 13b. Cement exhibits the greatest influence, while silty clay has the least impact on the elastic modulus of BPC, as depicted in Fig. 13c. Furthermore, Fig. 14 shows the summary plot which demonstrates the influence the input features on output parameter. It shows the order of SHAP value for a specific feature in addition to the trend of the related variable. The vertical axis of the SHAP plot displays the variables used as inputs and their importance in decreasing order, while the x-axis displays each individual SHAP result. The dots are data instances, and the size of the dots is represented by their color, which goes from blue to red. The x-axis shows the value of the estimate for each feature's SHAP values as the input parameter's intensity changes (from blue to red). Each variable's high feature value indicates that it has a favorable impact on the output result, as given in Fig. 14. Nevertheless, the smaller the attribute value is, the greater the unfavorable influence of the input parameter on the output. As shown in Fig. 14a, a higher amount of water has favorable effects on the slump, while a higher amount of cement has negative impacts on the slump. It is noticeable from Fig. 14b that the high feature value of water has significantly unfavorable effects on the *fc* of BPC, while, on the other hand, gravel, cement, and curing have positive impacts on compressive strength. Similarly, higher amounts of cement and gravel have favorable effects on elastic modulus, as given in Fig. 14c.

## Conclusion

In the present study, the slump, *fc*, and *Ec* of BPC have been modeled using multi-expression programming. An extensive database of 158 datasets for the slump, 169 for compressive strength, and 111 for elastic modulus have been collected from the experimental studies available on BPC. The most influential input parameters are considered for MEP modeling. The large database has been divided into three distinct categories of training, testing, and validation with the purpose of well-training the model on unseen data. Various statistical parameters (R, MAE, RMSE, RSE, and RRMSE) have been utilized to check the predictive capability and performance of the MEP models. Furthermore, all three models have been validated by using various external validation criteria. SHAP analysis was conducted for all models to discover the impact of input parameters on the output property.

The MEP models exhibited excellent accuracy with a correlation coefficient (R) of 0.9999 for slump, 0.9831 for *fc*, and 0.9300 for *Ec*. In addition, the developed models predicted the slump with MAE values of 1.4175 for training, 0.4272 for testing, and 0.2262 for validation. Similarly, the MEP model for compressive strength exhibited MAE values of 0.2245, 0.1574, and 0.3146 for training, testing, and validation, respectively. Overall, the suggested MEP models demonstrated higher accuracy and lower errors, indicating their robust prediction performance in estimating the properties of bentonite plastic concrete. Moreover, the comparative analysis between MEP models and conventional linear and non-linear regression models revealed remarkable precision in the predictions of the proposed MEP models, surpassing the accuracy of traditional regression methods. SHAP analysis was conducted to investigate the influence of various influential input parameters such as bentonite content, curing time, gravel, sand, cement, water, and silty clay on the properties of BPC. Water, cement, and bentonite have a significant influence on slump, but silty clay and sand have less effect. Furthermore, the water parameter has the maximum influence on compressive strength while curing time and cement has the higher impact on elastic modulus. In summary, this study provides crucial insights for builders and designers, elucidating the significance of each constituent in the mix design of bentonite plastic concrete. The application of ML algorithms offers the capability to deliver prompt and precise early estimates of BPC properties, thus optimizing the efficiency of construction and design processes.

The robustness of the developed models is contingent upon adherence to the prescribed ranges of input parameters used in this study. Any deviation beyond these limits warrants comprehensive validation to ensure the reliability of model predictions. Moreover, it is highly recommended to augment the dataset with a wider range of samples to enrich the model's predictive capacity. The inclusion of diverse data points spanning various scenarios and conditions will significantly enhance the model's robustness and generalizability, ensuring more accurate predictions across different contexts. In addition, exploring the integration of advanced global optimization bio-inspired algorithms, such as the human felicity algorithm, artificial bee colony algorithm, and tunicate swarm algorithm, for fine-tuning model hyperparameters is recommended. This approach has the potential to enhance the hybrid model's accuracy and robustness significantly, leading to a more reliable prediction model. Finally, it is recommended to utilize additional post-hoc model interpretability techniques, such as Individual conditional expectation plots and partial dependence plots, to gain deeper insights into the influence of input parameters on the properties of bentonite plastic concrete.

### Supplementary Information

Supplementary Information.

## Data Availability

Data and codes are provided in supplementary information files.
