# Supplementary material for: Computational prediction of workability and mechanical properties of bentonite plastic concrete using multi-expression programming
Source: Sci Rep. 2024 Mar 13;14:6105. doi: 10.1038/s41598-024-56088-0 (PMC10937993; doi:10.1038/s41598-024-56088-0)
Supplement: Supplementary file 1 — Supplementary Information. [file 41598_2024_56088_MOESM1_ESM.docx]

**Supplementary materials**

**The dataset used in this study has been collected from the Amlashi et al. study (**[**https://doi.org/10.1016/j.jclepro.2019.05.168**](https://doi.org/10.1016/j.jclepro.2019.05.168)**)**

**Table S1. Dataset used for slump prediction**

| Gravel | Sand | Silty Clay | Cement | Bentonite | Water | Slump |
| --- | --- | --- | --- | --- | --- | --- |
| 735 | 441 | 0 | 147 | 56 | 294 | 200 |
| 735 | 630.5 | 0 | 105 | 34 | 210 | 200 |
| 195 | 1305 | 0 | 100 | 30 | 420 | 190 |
| 195 | 1305 | 0 | 150 | 30 | 420 | 210 |
| 195 | 1305 | 0 | 150 | 40 | 420 | 220 |
| 195 | 1305 | 0 | 200 | 30 | 420 | 170 |
| 195 | 1305 | 0 | 200 | 40 | 420 | 200 |
| 195 | 1305 | 0 | 200 | 50 | 420 | 230 |
| 795 | 704.37 | 0 | 230 | 35 | 330 | 185 |
| 795 | 704.37 | 0 | 230 | 30 | 330 | 190 |
| 795 | 704.37 | 0 | 230 | 25 | 330 | 200 |
| 795 | 698.56 | 0 | 230 | 30 | 330 | 190 |
| 795 | 705 | 0 | 220 | 40 | 400 | 220 |
| 795 | 700 | 0 | 230 | 30 | 330 | 180 |
| 795 | 705 | 0 | 220 | 35 | 340 | 180 |
| 795 | 700 | 0 | 220 | 35 | 350 | 180 |
| 795 | 705 | 0 | 220 | 38 | 335 | 180 |
| 795 | 700 | 0 | 220 | 37 | 335 | 180 |
| 795 | 705 | 0 | 220 | 37.5 | 340 | 185 |
| 795 | 705 | 0 | 200 | 39 | 330 | 180 |
| 795 | 705 | 0 | 195 | 39 | 330 | 180 |
| 795 | 705 | 0 | 205 | 39 | 330 | 180 |
| 795 | 705 | 0 | 210 | 39 | 330 | 180 |
| 795 | 705 | 0 | 215 | 39 | 330 | 180 |
| 795 | 705 | 0 | 210 | 40 | 330 | 180 |
| 795 | 705 | 0 | 205 | 39 | 330 | 180 |
| 800 | 720 | 0 | 205 | 37 | 325 | 180 |
| 800 | 720 | 0 | 200 | 37 | 325 | 180 |
| 800 | 700 | 0 | 120 | 40 | 320 | 200 |
| 800 | 700 | 0 | 120 | 39 | 330 | 200 |
| 800 | 700 | 0 | 110 | 39 | 330 | 200 |
| 800 | 700 | 0 | 120 | 39 | 330 | 200 |
| 800 | 700 | 0 | 100 | 39 | 330 | 200 |
| 800 | 720 | 0 | 205 | 37 | 325 | 200 |
| 827 | 676 | 0 | 192 | 31.4 | 345 | 150 |
| 622 | 509 | 0 | 258 | 42.3 | 465 | 230 |
| 645 | 788 | 0 | 203 | 33.2 | 365 | 145 |
| 467 | 571 | 0 | 274 | 44.8 | 493 | 225 |
| 0 | 1372 | 0 | 214 | 35 | 385 | 145 |
| 0 | 955 | 0 | 289 | 47.3 | 520 | 225 |
| 926 | 757 | 0 | 115 | 27.3 | 300 | 153 |
| 755 | 617 | 0 | 156 | 36.8 | 405 | 210 |
| 708 | 865 | 0 | 129 | 30.5 | 335 | 150 |
| 552 | 675 | 0 | 174 | 41.1 | 452 | 214 |
| 0 | 1499 | 0 | 138 | 32.7 | 360 | 153 |
| 0 | 1128 | 0 | 187 | 44.2 | 486 | 215 |
| 795 | 705 | 0 | 220 | 30.8 | 349.8 | 175 |
| 795 | 705 | 0 | 220 | 35.2 | 349.8 | 170 |
| 795 | 705 | 0 | 220 | 37.4 | 349.8 | 190 |
| 795 | 705 | 0 | 220 | 39.6 | 400.4 | 220 |
| 795 | 705 | 0 | 200 | 40 | 350 | 180 |
| 795 | 705 | 0 | 210 | 39.9 | 350.7 | 180 |
| 790 | 705 | 0 | 200 | 38 | 350 | 180 |
| 800 | 700 | 0 | 120 | 39.6 | 350.4 | 180 |
| 800 | 700 | 0 | 150 | 22.5 | 298.5 | 190 |
| 800 | 700 | 0 | 150 | 37.5 | 354 | 190 |
| 800 | 700 | 0 | 150 | 52.5 | 390 | 185 |
| 800 | 700 | 0 | 200 | 30 | 312 | 195 |
| 800 | 700 | 0 | 200 | 50 | 376 | 200 |
| 800 | 700 | 0 | 200 | 70 | 412 | 190 |
| 450 | 900 | 150 | 120 | 48 | 300 | 165 |
| 450 | 900 | 150 | 120 | 32 | 300 | 210 |
| 450 | 900 | 150 | 120 | 16 | 300 | 220 |
| 909 | 775 | 0 | 180 | 25 | 288 | 180 |
| 857 | 823 | 0 | 220 | 22 | 286 | 190 |
| 888 | 726 | 0 | 250 | 20.33 | 300 | 190 |
| 834 | 682 | 0 | 250 | 20 | 325 | 190 |
| 889 | 728 | 0 | 250 | 20 | 300 | 190 |
| 889 | 728 | 0 | 250 | 20 | 300 | 200 |
| 912 | 746 | 0 | 200 | 20 | 300 | 200 |
| 912 | 778 | 0 | 200 | 20 | 290 | 200 |
| 844 | 811 | 0 | 220 | 24 | 294.8 | 190 |
| 823 | 823 | 0 | 100 | 25 | 330 | 190 |
| 811 | 811 | 0 | 130 | 25 | 330 | 190 |
| 799 | 798 | 0 | 160 | 25 | 330 | 185 |
| 786 | 786 | 0 | 190 | 25 | 330 | 185 |
| 774 | 774 | 0 | 220 | 25 | 330 | 180 |
| 809 | 809 | 0 | 100 | 40 | 330 | 190 |
| 802 | 803 | 0 | 130 | 40 | 330 | 185 |
| 790 | 790 | 0 | 160 | 40 | 330 | 185 |
| 778 | 778 | 0 | 190 | 40 | 330 | 180 |
| 766 | 766 | 0 | 220 | 40 | 330 | 180 |
| 726 | 726 | 0 | 100 | 55 | 395 | 205 |
| 714 | 714 | 0 | 130 | 55 | 395 | 200 |
| 702 | 702 | 0 | 160 | 55 | 395 | 195 |
| 689 | 689 | 0 | 190 | 55 | 395 | 195 |
| 677 | 677 | 0 | 220 | 55 | 395 | 190 |
| 750 | 750 | 0 | 200 | 30 | 300 | 75 |
| 750 | 750 | 0 | 200 | 30 | 400 | 200 |
| 750 | 750 | 0 | 200 | 30 | 500 | 220 |
| 750 | 750 | 0 | 200 | 40 | 300 | 65 |
| 750 | 750 | 0 | 200 | 40 | 400 | 170 |
| 750 | 750 | 0 | 200 | 40 | 500 | 220 |
| 750 | 750 | 0 | 200 | 50 | 300 | 45 |
| 750 | 750 | 0 | 200 | 50 | 400 | 180 |
| 750 | 750 | 0 | 200 | 50 | 500 | 200 |
| 850 | 750 | 0 | 150 | 36 | 450 | 210 |
| 795 | 705 | 0 | 195 | 37 | 360 | 190 |
| 750 | 750 | 0 | 200 | 25 | 280 | 45 |
| 750 | 750 | 0 | 200 | 20 | 240 | 40 |
| 750 | 750 | 0 | 200 | 15 | 180 | 15 |
| 750 | 750 | 0 | 225 | 30 | 315 | 55 |
| 750 | 750 | 0 | 225 | 25 | 270 | 30 |
| 750 | 750 | 0 | 225 | 20 | 202.5 | 8 |
| 750 | 750 | 0 | 250 | 30 | 350 | 61 |
| 750 | 750 | 0 | 250 | 25 | 300 | 45 |
| 750 | 750 | 0 | 250 | 20 | 225 | 10 |
| 750 | 750 | 0 | 275 | 35 | 385 | 100 |
| 750 | 750 | 0 | 275 | 30 | 330 | 55 |
| 750 | 750 | 0 | 275 | 20 | 247.5 | 25 |
| 750 | 750 | 0 | 300 | 35 | 420 | 110 |
| 750 | 750 | 0 | 300 | 30 | 360 | 65 |
| 666.25 | 666.25 | 180 | 150 | 80 | 307.5 | 180 |
| 641.65 | 641.65 | 180 | 150 | 80 | 356.7 | 200 |
| 625 | 625 | 180 | 150 | 70 | 400 | 240 |
| 786 | 524 | 180 | 120 | 70 | 370 | 230 |
| 655 | 655 | 180 | 120 | 70 | 370 | 230 |
| 524 | 786 | 180 | 120 | 70 | 370 | 230 |
| 685 | 685 | 180 | 90 | 70 | 340 | 210 |
| 655 | 655 | 180 | 120 | 70 | 322 | 205 |
| 641.5 | 641.5 | 220 | 120 | 70 | 357 | 210 |
| 604.5 | 604.5 | 260 | 120 | 70 | 391 | 210 |
| 707 | 707 | 180 | 120 | 40 | 296 | 200 |
| 651 | 651 | 180 | 120 | 100 | 348 | 220 |
| 860 | 790 | 0 | 200 | 15 | 260 | 190 |
| 860 | 790 | 0 | 200 | 20 | 300 | 200 |
| 860 | 790 | 0 | 200 | 25 | 340 | 190 |
| 860 | 790 | 0 | 200 | 30 | 375 | 180 |
| 860 | 790 | 0 | 200 | 35 | 420 | 170 |
| 750 | 750 | 0 | 150 | 32 | 400 | 215 |
| 750 | 750 | 0 | 150 | 36 | 400 | 210 |
| 750 | 750 | 0 | 150 | 40 | 400 | 210 |
| 750 | 750 | 0 | 150 | 44 | 400 | 210 |
| 750 | 750 | 0 | 150 | 48 | 400 | 210 |
| 775 | 775 | 0 | 252 | 28 | 190.4 | 200 |
| 775 | 775 | 0 | 224 | 56 | 212.8 | 200 |
| 775 | 775 | 0 | 196 | 84 | 219.67 | 200 |
| 775 | 775 | 0 | 168 | 112 | 252 | 200 |
| 775 | 775 | 0 | 140 | 140 | 281.04 | 200 |
| 775 | 775 | 0 | 112 | 168 | 302.4 | 200 |
| 295 | 1305 | 205 | 252 | 28 | 349.72 | 200 |
| 295 | 1305 | 205 | 224 | 56 | 361.63 | 200 |
| 295 | 1305 | 205 | 196 | 84 | 390.54 | 200 |
| 295 | 1305 | 205 | 168 | 112 | 410.78 | 200 |
| 295 | 1305 | 205 | 140 | 140 | 429.95 | 200 |
| 295 | 1305 | 205 | 112 | 168 | 481.47 | 200 |
| 310 | 1290 | 225 | 180 | 20 | 340.48 | 200 |
| 310 | 1290 | 225 | 160 | 40 | 356.79 | 200 |
| 310 | 1290 | 225 | 140 | 60 | 375.36 | 200 |
| 310 | 1290 | 225 | 120 | 80 | 395.78 | 200 |
| 310 | 1290 | 225 | 100 | 100 | 405.16 | 200 |
| 310 | 1290 | 225 | 80 | 120 | 413.17 | 200 |
| 875 | 875 | 0 | 162 | 18 | 152.1 | 200 |
| 875 | 875 | 0 | 144 | 36 | 162 | 200 |
| 875 | 875 | 0 | 126 | 54 | 166.67 | 200 |
| 875 | 875 | 0 | 108 | 72 | 190.8 | 200 |
| 875 | 875 | 0 | 90 | 90 | 220.8 | 200 |
| 875 | 875 | 0 | 72 | 108 | 243 | 200 |

**Table S2. Dataset used for compressive strength prediction**

| Gravel | Sand | Clay | Cement | Bentonit | Water | Curing time | fc |
| --- | --- | --- | --- | --- | --- | --- | --- |
| 875 | 875 | 0 | 162 | 18 | 152.1 | 7 | 12.75 |
| 875 | 875 | 0 | 144 | 36 | 162 | 7 | 8.24 |
| 875 | 875 | 0 | 126 | 54 | 166.67 | 7 | 6.08 |
| 875 | 875 | 0 | 108 | 72 | 190.8 | 7 | 3.63 |
| 875 | 875 | 0 | 90 | 90 | 220.8 | 7 | 2.16 |
| 875 | 875 | 0 | 72 | 108 | 243 | 7 | 1.37 |
| 875 | 875 | 0 | 162 | 18 | 152.1 | 28 | 14.72 |
| 875 | 875 | 0 | 144 | 36 | 162 | 28 | 9.9 |
| 875 | 875 | 0 | 126 | 54 | 166.67 | 28 | 6.97 |
| 875 | 875 | 0 | 108 | 72 | 190.8 | 28 | 4.71 |
| 875 | 875 | 0 | 90 | 90 | 220.8 | 28 | 3.73 |
| 875 | 875 | 0 | 72 | 108 | 243 | 28 | 2.35 |
| 875 | 875 | 0 | 162 | 18 | 152.1 | 90 | 21.78 |
| 875 | 875 | 0 | 144 | 36 | 162 | 90 | 14.91 |
| 875 | 875 | 0 | 126 | 54 | 166.67 | 90 | 13.05 |
| 875 | 875 | 0 | 108 | 72 | 190.8 | 90 | 8.53 |
| 875 | 875 | 0 | 90 | 90 | 220.8 | 90 | 6.67 |
| 875 | 875 | 0 | 72 | 108 | 243 | 90 | 3.24 |
| 820 | 750 | 140 | 120 | 35 | 340 | 28 | 2.17 |
| 820 | 750 | 140 | 120 | 35 | 340 | 14 | 2.24 |
| 810 | 710 | 160 | 120 | 50 | 350 | 28 | 3.1 |
| 810 | 710 | 160 | 120 | 50 | 350 | 90 | 4.27 |
| 786 | 524 | 180 | 120 | 70 | 370 | 28 | 1.1 |
| 786 | 524 | 180 | 120 | 70 | 370 | 90 | 2.02 |
| 786 | 524 | 180 | 120 | 70 | 370 | 28 | 1.3 |
| 786 | 524 | 180 | 120 | 70 | 370 | 90 | 1.91 |
| 786 | 524 | 180 | 120 | 70 | 370 | 540 | 3.2 |
| 775 | 775 | 0 | 252 | 28 | 190.4 | 7 | 10.79 |
| 775 | 775 | 0 | 224 | 56 | 212.8 | 7 | 9.81 |
| 775 | 775 | 0 | 196 | 84 | 219.67 | 7 | 7.85 |
| 775 | 775 | 0 | 168 | 112 | 252 | 7 | 5.79 |
| 775 | 775 | 0 | 140 | 140 | 281.04 | 7 | 4.02 |
| 775 | 775 | 0 | 112 | 168 | 302.4 | 7 | 2.75 |
| 775 | 775 | 0 | 252 | 28 | 190.4 | 28 | 12.26 |
| 775 | 775 | 0 | 224 | 56 | 212.8 | 28 | 10.69 |
| 775 | 775 | 0 | 196 | 84 | 219.67 | 28 | 9.42 |
| 775 | 775 | 0 | 168 | 112 | 252 | 28 | 8.14 |
| 775 | 775 | 0 | 140 | 140 | 281.04 | 28 | 5.59 |
| 775 | 775 | 0 | 112 | 168 | 302.4 | 28 | 3.53 |
| 775 | 775 | 0 | 252 | 28 | 190.4 | 90 | 20.99 |
| 775 | 775 | 0 | 224 | 56 | 212.8 | 90 | 19.12 |
| 775 | 775 | 0 | 196 | 84 | 219.67 | 90 | 13.73 |
| 775 | 775 | 0 | 168 | 112 | 252 | 90 | 9.41 |
| 775 | 775 | 0 | 140 | 140 | 281.04 | 90 | 7.36 |
| 775 | 775 | 0 | 112 | 168 | 302.4 | 90 | 5 |
| 760 | 660 | 230 | 120 | 50 | 380 | 28 | 2.74 |
| 760 | 660 | 230 | 120 | 50 | 380 | 90 | 3.8 |
| 756 | 684 | 210 | 100 | 50 | 400 | 28 | 2.97 |
| 756 | 684 | 210 | 100 | 50 | 400 | 14 | 1.85 |
| 740 | 650 | 270 | 120 | 50 | 370 | 28 | 3.18 |
| 740 | 650 | 270 | 120 | 50 | 370 | 28 | 3.18 |
| 740 | 650 | 270 | 120 | 50 | 370 | 90 | 4.64 |
| 737 | 663 | 230 | 50 | 70 | 450 | 28 | 1.11 |
| 737 | 663 | 230 | 50 | 70 | 450 | 14 | 0.8 |
| 734 | 661 | 230 | 65 | 70 | 450 | 28 | 1.56 |
| 734 | 661 | 230 | 65 | 70 | 450 | 14 | 1.4 |
| 730 | 640 | 280 | 80 | 20 | 450 | 28 | 2.19 |
| 710 | 620 | 280 | 120 | 20 | 450 | 28 | 2.7 |
| 710 | 620 | 310 | 120 | 50 | 390 | 28 | 3.22 |
| 707 | 707 | 180 | 120 | 40 | 296 | 28 | 2.47 |
| 707 | 707 | 180 | 120 | 40 | 296 | 90 | 3.07 |
| 707 | 707 | 180 | 120 | 40 | 296 | 28 | 2.47 |
| 707 | 707 | 180 | 120 | 40 | 296 | 90 | 2.91 |
| 707 | 707 | 180 | 120 | 40 | 296 | 540 | 4.3 |
| 700 | 620 | 310 | 120 | 50 | 400 | 28 | 2.57 |
| 700 | 620 | 310 | 120 | 50 | 400 | 90 | 3.6 |
| 690 | 600 | 280 | 160 | 20 | 450 | 28 | 3.7 |
| 690 | 610 | 330 | 120 | 50 | 400 | 28 | 1.8 |
| 685 | 600 | 350 | 120 | 50 | 395 | 28 | 3.17 |
| 685 | 685 | 180 | 90 | 70 | 340 | 28 | 1.29 |
| 685 | 685 | 180 | 90 | 70 | 340 | 90 | 1.74 |
| 685 | 685 | 180 | 90 | 70 | 340 | 28 | 1.27 |
| 685 | 685 | 180 | 90 | 70 | 340 | 90 | 1.71 |
| 685 | 685 | 180 | 90 | 70 | 340 | 540 | 1.9 |
| 670 | 590 | 0 | 120 | 320 | 500 | 28 | 2.63 |
| 666.25 | 666.25 | 180 | 150 | 80 | 307.5 | 28 | 3.27 |
| 666.25 | 666.25 | 180 | 150 | 80 | 307.5 | 90 | 4.59 |
| 666.25 | 666.25 | 180 | 150 | 80 | 307.5 | 180 | 5.63 |
| 666.25 | 666.25 | 180 | 150 | 80 | 307.5 | 28 | 3.26 |
| 666.25 | 666.25 | 180 | 150 | 80 | 307.5 | 90 | 4.26 |
| 666.25 | 666.25 | 180 | 150 | 80 | 307.5 | 540 | 5.4 |
| 660 | 590 | 380 | 120 | 50 | 400 | 28 | 3.13 |
| 655 | 655 | 180 | 120 | 70 | 370 | 28 | 1.5 |
| 655 | 655 | 180 | 120 | 70 | 370 | 90 | 2.15 |
| 655 | 655 | 180 | 120 | 70 | 322 | 28 | 2.31 |
| 655 | 655 | 180 | 120 | 70 | 322 | 90 | 2.72 |
| 655 | 655 | 180 | 120 | 70 | 370 | 28 | 1.56 |
| 655 | 655 | 180 | 120 | 70 | 322 | 28 | 1.98 |
| 655 | 655 | 180 | 120 | 70 | 370 | 90 | 2.09 |
| 655 | 655 | 180 | 120 | 70 | 322 | 90 | 2.72 |
| 655 | 655 | 180 | 120 | 70 | 370 | 540 | 3.5 |
| 655 | 655 | 180 | 120 | 70 | 322 | 540 | 4.4 |
| 651 | 651 | 180 | 120 | 100 | 348 | 28 | 1.79 |
| 651 | 651 | 180 | 120 | 100 | 348 | 90 | 2.48 |
| 651 | 651 | 180 | 120 | 100 | 348 | 28 | 1.64 |
| 651 | 651 | 180 | 120 | 100 | 348 | 90 | 2.52 |
| 651 | 651 | 180 | 120 | 100 | 348 | 540 | 4 |
| 641.65 | 641.65 | 180 | 150 | 80 | 356.7 | 28 | 2.37 |
| 641.65 | 641.65 | 180 | 150 | 80 | 356.7 | 90 | 3.01 |
| 641.65 | 641.65 | 180 | 150 | 80 | 356.7 | 180 | 4.14 |
| 641.65 | 641.65 | 180 | 150 | 80 | 356.7 | 28 | 2.01 |
| 641.65 | 641.65 | 180 | 150 | 80 | 356.7 | 90 | 2.92 |
| 641.65 | 641.65 | 180 | 150 | 80 | 356.7 | 540 | 4.8 |
| 641.5 | 641.5 | 220 | 120 | 70 | 357 | 28 | 1.94 |
| 641.5 | 641.5 | 220 | 120 | 70 | 357 | 90 | 2.63 |
| 641.5 | 641.5 | 220 | 120 | 70 | 357 | 28 | 1.82 |
| 641.5 | 641.5 | 220 | 120 | 70 | 357 | 90 | 2.51 |
| 641.5 | 641.5 | 220 | 120 | 70 | 357 | 540 | 4 |
| 625 | 625 | 180 | 150 | 70 | 400 | 28 | 1.68 |
| 625 | 625 | 180 | 150 | 70 | 400 | 90 | 2.68 |
| 625 | 625 | 180 | 150 | 70 | 400 | 180 | 2.88 |
| 625 | 625 | 180 | 150 | 70 | 400 | 28 | 1.62 |
| 625 | 625 | 180 | 150 | 80 | 400 | 90 | 2.12 |
| 625 | 625 | 180 | 150 | 80 | 400 | 540 | 3.1 |
| 604.5 | 604.5 | 260 | 120 | 70 | 391 | 28 | 1.27 |
| 604.5 | 604.5 | 260 | 120 | 70 | 391 | 90 | 2.11 |
| 604.5 | 604.5 | 260 | 120 | 70 | 391 | 28 | 1.32 |
| 604.5 | 604.5 | 260 | 120 | 70 | 391 | 90 | 1.97 |
| 604.5 | 604.5 | 260 | 120 | 70 | 391 | 540 | 2.9 |
| 524 | 786 | 180 | 120 | 70 | 370 | 28 | 1.81 |
| 524 | 786 | 180 | 120 | 70 | 370 | 90 | 2.3 |
| 524 | 786 | 180 | 120 | 70 | 370 | 28 | 1.39 |
| 524 | 786 | 180 | 120 | 70 | 370 | 90 | 2.04 |
| 524 | 786 | 180 | 120 | 70 | 370 | 540 | 3 |
| 450 | 900 | 150 | 120 | 48 | 300 | 7 | 1.3 |
| 450 | 900 | 150 | 120 | 32 | 300 | 7 | 1.13 |
| 450 | 900 | 150 | 120 | 16 | 300 | 7 | 1.01 |
| 450 | 900 | 150 | 120 | 48 | 300 | 28 | 1.77 |
| 450 | 900 | 150 | 120 | 32 | 300 | 28 | 1.5 |
| 450 | 900 | 150 | 120 | 16 | 300 | 28 | 1.4 |
| 450 | 900 | 150 | 120 | 48 | 300 | 90 | 2.12 |
| 450 | 900 | 150 | 120 | 32 | 300 | 90 | 2.03 |
| 450 | 900 | 150 | 120 | 16 | 300 | 90 | 1.5 |
| 310 | 1290 | 225 | 180 | 20 | 340.48 | 7 | 2.26 |
| 310 | 1290 | 225 | 160 | 40 | 356.79 | 7 | 1.86 |
| 310 | 1290 | 225 | 140 | 60 | 375.36 | 7 | 1.47 |
| 310 | 1290 | 225 | 120 | 80 | 395.78 | 7 | 1.08 |
| 310 | 1290 | 225 | 100 | 100 | 405.16 | 7 | 0.98 |
| 310 | 1290 | 225 | 80 | 120 | 413.17 | 7 | 0.98 |
| 310 | 1290 | 225 | 180 | 20 | 340.48 | 28 | 3.14 |
| 310 | 1290 | 225 | 160 | 40 | 356.79 | 28 | 2.75 |
| 310 | 1290 | 225 | 140 | 60 | 375.36 | 28 | 2.55 |
| 310 | 1290 | 225 | 120 | 80 | 395.78 | 28 | 2.26 |
| 310 | 1290 | 225 | 100 | 100 | 405.16 | 28 | 2.16 |
| 310 | 1290 | 225 | 80 | 120 | 413.17 | 28 | 2.16 |
| 310 | 1290 | 225 | 180 | 20 | 340.48 | 90 | 4.81 |
| 310 | 1290 | 225 | 160 | 40 | 356.79 | 90 | 4.51 |
| 310 | 1290 | 225 | 140 | 60 | 375.36 | 90 | 3.83 |
| 310 | 1290 | 225 | 120 | 80 | 395.78 | 90 | 3.34 |
| 310 | 1290 | 225 | 100 | 100 | 405.16 | 90 | 2.65 |
| 310 | 1290 | 225 | 80 | 120 | 413.17 | 90 | 2.35 |
| 295 | 1305 | 205 | 252 | 28 | 349.72 | 7 | 4.41 |
| 295 | 1305 | 205 | 224 | 56 | 361.63 | 7 | 3.24 |
| 295 | 1305 | 205 | 196 | 84 | 390.54 | 7 | 2.35 |
| 295 | 1305 | 205 | 168 | 112 | 410.78 | 7 | 1.57 |
| 295 | 1305 | 205 | 140 | 140 | 429.95 | 7 | 1.18 |
| 295 | 1305 | 205 | 112 | 168 | 481.47 | 7 | 0.98 |
| 295 | 1305 | 205 | 252 | 28 | 349.72 | 28 | 7.36 |
| 295 | 1305 | 205 | 224 | 56 | 361.63 | 28 | 5.49 |
| 295 | 1305 | 205 | 196 | 84 | 390.54 | 28 | 4.22 |
| 295 | 1305 | 205 | 168 | 112 | 410.78 | 28 | 2.84 |
| 295 | 1305 | 205 | 140 | 140 | 429.95 | 28 | 2.35 |
| 295 | 1305 | 205 | 112 | 168 | 481.47 | 28 | 2.06 |
| 295 | 1305 | 205 | 252 | 28 | 349.72 | 90 | 9.81 |
| 295 | 1305 | 205 | 224 | 56 | 361.63 | 90 | 7.46 |
| 295 | 1305 | 205 | 196 | 84 | 390.54 | 90 | 5.89 |
| 295 | 1305 | 205 | 168 | 112 | 410.78 | 90 | 4.41 |
| 295 | 1305 | 205 | 140 | 140 | 429.95 | 90 | 3.43 |
| 295 | 1305 | 205 | 112 | 168 | 481.47 | 90 | 2.75 |

**Table S3. Dataset used for elastic modulus prediction**

| Curing time | Water | Bentonit | Cement | Silty clay | Sand | Gravel | Ec |
| --- | --- | --- | --- | --- | --- | --- | --- |
| 7 | 330 | 35 | 230 | 0 | 704.37 | 795 | 216.34 |
| 7 | 330 | 30 | 230 | 0 | 704.37 | 795 | 236.47 |
| 7 | 330 | 25 | 230 | 0 | 704.37 | 795 | 313.5 |
| 7 | 330 | 30 | 230 | 0 | 698.56 | 795 | 557.24 |
| 28 | 330 | 35 | 230 | 0 | 704.37 | 795 | 392.29 |
| 28 | 330 | 30 | 230 | 0 | 704.37 | 795 | 738.39 |
| 28 | 330 | 25 | 230 | 0 | 704.37 | 795 | 838.41 |
| 28 | 330 | 30 | 230 | 0 | 698.56 | 795 | 852.36 |
| 42 | 330 | 35 | 230 | 0 | 704.37 | 795 | 777.94 |
| 42 | 330 | 30 | 230 | 0 | 704.37 | 795 | 828.2 |
| 42 | 330 | 25 | 230 | 0 | 704.37 | 795 | 1048.47 |
| 42 | 330 | 30 | 230 | 0 | 698.56 | 795 | 1239.29 |
| 90 | 330 | 35 | 230 | 0 | 704.37 | 795 | 1447.76 |
| 90 | 330 | 30 | 230 | 0 | 704.37 | 795 | 2053.07 |
| 90 | 330 | 25 | 230 | 0 | 704.37 | 795 | 2224.16 |
| 90 | 330 | 30 | 230 | 0 | 698.56 | 795 | 2592.03 |
| 90 | 345 | 31.4 | 192 | 0 | 676 | 827 | 7596.86 |
| 90 | 465 | 42.3 | 258 | 0 | 509 | 622 | 4132.95 |
| 90 | 365 | 33.2 | 203 | 0 | 788 | 645 | 7822.49 |
| 90 | 493 | 44.8 | 274 | 0 | 571 | 467 | 4379.18 |
| 90 | 385 | 35 | 214 | 0 | 1372 | 0 | 6583.49 |
| 90 | 520 | 47.3 | 289 | 0 | 955 | 0 | 3741.53 |
| 90 | 300 | 27.3 | 115 | 0 | 757 | 926 | 3519.83 |
| 90 | 405 | 36.8 | 156 | 0 | 617 | 755 | 2392.169 |
| 90 | 335 | 30.5 | 129 | 0 | 865 | 708 | 3581.63 |
| 90 | 452 | 41.1 | 174 | 0 | 675 | 552 | 2490.76 |
| 90 | 360 | 32.7 | 138 | 0 | 1499 | 0 | 3037.18 |
| 90 | 486 | 44.2 | 187 | 0 | 1128 | 0 | 2108.17 |
| 28 | 400 | 50 | 120 | 370 | 590 | 670 | 1671 |
| 28 | 400 | 50 | 120 | 310 | 620 | 700 | 1344 |
| 28 | 350 | 50 | 120 | 190 | 700 | 790 | 1090 |
| 28 | 380 | 50 | 120 | 330 | 620 | 700 | 1255 |
| 28 | 380 | 50 | 120 | 230 | 660 | 760 | 1354 |
| 28 | 390 | 50 | 120 | 310 | 620 | 710 | 1100 |
| 28 | 450 | 20 | 80 | 280 | 640 | 730 | 848 |
| 28 | 450 | 20 | 120 | 280 | 620 | 710 | 964 |
| 28 | 450 | 20 | 160 | 280 | 600 | 690 | 1431 |
| 28 | 395 | 50 | 120 | 350 | 600 | 685 | 1070 |
| 28 | 420 | 42 | 280 | 0 | 1294 | 1519.6 | 679 |
| 28 | 418.5 | 46.5 | 280 | 0 | 1294 | 1519.6 | 678 |
| 28 | 375 | 37.5 | 250 | 0 | 1294 | 1519.6 | 905 |
| 28 | 375.3 | 41.7 | 250 | 0 | 1294 | 1519.6 | 806 |
| 28 | 374 | 34 | 250 | 0 | 1351 | 1463 | 750 |
| 28 | 282.6 | 31.4 | 230 | 0 | 1351 | 1463 | 780 |
| 28 | 375 | 37.5 | 250 | 0 | 1294 | 1519.6 | 980 |
| 28 | 360 | 40 | 330 | 0 | 1294 | 1519.6 | 1590 |
| 28 | 360 | 40 | 300 | 0 | 1294 | 1519.6 | 1224 |
| 28 | 389.7 | 43.3 | 300 | 0 | 1294 | 1519.6 | 1325 |
| 28 | 349.2 | 38.8 | 255 | 0 | 1294 | 1519.6 | 1327 |
| 28 | 295 | 14.8 | 250 | 0 | 1266 | 1547.7 | 1905 |
| 28 | 495 | 66 | 160 | 0 | 1351 | 1463 | 610 |
| 28 | 726 | 66 | 160 | 0 | 1351 | 1463 | 837 |
| 28 | 330 | 66 | 160 | 0 | 1351 | 1463 | 934 |
| 28 | 304 | 60.8 | 160 | 0 | 1351 | 1463 | 790 |
| 28 | 330 | 30 | 160 | 0 | 1351 | 1463 | 717 |
| 90 | 330 | 25 | 100 | 0 | 823 | 823 | 1307.67 |
| 90 | 330 | 25 | 130 | 0 | 811 | 811 | 2869.43 |
| 90 | 330 | 25 | 160 | 0 | 798 | 799 | 3955.98 |
| 90 | 330 | 25 | 190 | 0 | 786 | 786 | 5205.19 |
| 90 | 330 | 25 | 220 | 0 | 774 | 774 | 6874.36 |
| 90 | 330 | 40 | 100 | 0 | 809 | 809 | 1146.59 |
| 90 | 330 | 40 | 130 | 0 | 803 | 802 | 2693.63 |
| 90 | 330 | 40 | 160 | 0 | 790 | 790 | 3723.88 |
| 90 | 330 | 40 | 190 | 0 | 778 | 778 | 4770.9 |
| 90 | 330 | 40 | 220 | 0 | 766 | 766 | 6048.55 |
| 90 | 395 | 55 | 100 | 0 | 726 | 726 | 812.27 |
| 90 | 395 | 55 | 130 | 0 | 714 | 714 | 1277.75 |
| 90 | 395 | 55 | 160 | 0 | 702 | 702 | 2095.81 |
| 90 | 395 | 55 | 190 | 0 | 689 | 689 | 3201.69 |
| 90 | 395 | 55 | 220 | 0 | 677 | 677 | 4154.54 |
| 28 | 300 | 48 | 120 | 150 | 900 | 450 | 97.15 |
| 28 | 300 | 32 | 120 | 150 | 900 | 450 | 135.77 |
| 28 | 300 | 16 | 120 | 150 | 900 | 450 | 116.98 |
| 28 | 400 | 50 | 120 | 270 | 720 | 640 | 1118 |
| 28 | 390 | 50 | 120 | 310 | 710 | 620 | 1100 |
| 28 | 400 | 50 | 120 | 330 | 690 | 610 | 1084 |
| 28 | 395 | 50 | 120 | 350 | 685 | 600 | 1069 |
| 28 | 400 | 50 | 120 | 370 | 670 | 590 | 1050 |
| 28 | 400 | 50 | 120 | 380 | 660 | 590 | 1040 |
| 28 | 307.5 | 80 | 150 | 180 | 666.25 | 666.25 | 1455.57 |
| 28 | 356.7 | 80 | 150 | 180 | 641.65 | 641.65 | 808.2 |
| 28 | 400 | 80 | 150 | 180 | 625 | 625 | 745.35 |
| 90 | 307.5 | 80 | 150 | 180 | 666.25 | 666.25 | 2078.17 |
| 90 | 356.7 | 80 | 150 | 180 | 641.65 | 641.65 | 1452.43 |
| 90 | 400 | 80 | 150 | 180 | 625 | 625 | 1328.37 |
| 180 | 307.5 | 80 | 150 | 180 | 666.25 | 666.25 | 3152.37 |
| 180 | 356.7 | 80 | 150 | 180 | 641.65 | 641.65 | 1740.13 |
| 180 | 400 | 80 | 150 | 180 | 625 | 625 | 1051.26 |
| 28 | 370 | 70 | 120 | 180 | 524 | 786 | 582.83 |
| 28 | 370 | 70 | 120 | 180 | 655 | 655 | 868.17 |
| 28 | 370 | 70 | 120 | 180 | 786 | 524 | 500.47 |
| 90 | 370 | 70 | 120 | 180 | 524 | 786 | 1049.6 |
| 90 | 370 | 70 | 120 | 180 | 655 | 655 | 1308.8 |
| 90 | 370 | 70 | 120 | 180 | 786 | 524 | 1229.17 |
| 28 | 340 | 70 | 90 | 180 | 685 | 685 | 497.93 |
| 90 | 340 | 70 | 90 | 180 | 685 | 685 | 741.73 |
| 28 | 322 | 70 | 120 | 180 | 655 | 655 | 1148 |
| 28 | 357 | 70 | 120 | 220 | 641.5 | 641.5 | 966.6667 |
| 28 | 391 | 70 | 120 | 260 | 604.5 | 604.5 | 589.6 |
| 90 | 322 | 70 | 120 | 180 | 655 | 655 | 1667.87 |
| 90 | 357 | 70 | 120 | 220 | 641.5 | 641.5 | 1150.63 |
| 90 | 391 | 70 | 120 | 260 | 604.5 | 604.5 | 992.52 |
| 28 | 296 | 40 | 120 | 180 | 707 | 707 | 1233.3 |
| 28 | 348 | 100 | 120 | 180 | 651 | 651 | 738.03 |
| 90 | 296 | 40 | 120 | 180 | 707 | 707 | 1476.1 |
| 90 | 348 | 100 | 120 | 180 | 651 | 651 | 1477.13 |
| 28 | 260 | 15 | 200 | 0 | 790 | 860 | 2570.22 |
| 28 | 300 | 20 | 200 | 0 | 790 | 860 | 2020.86 |
| 28 | 340 | 25 | 200 | 0 | 790 | 860 | 1494.16 |
| 28 | 375 | 30 | 200 | 0 | 790 | 860 | 931.46 |
| 28 | 420 | 35 | 200 | 0 | 790 | 860 | 540.63 |

**Table S4.** MEP generated code for slump prediction

| #include <math.h>  #include <stdio.h>  void mepx(double *x /*inputs*/, double *outputs)  {  double prg[50];  prg[0] = x[1];  prg[1] = x[6];  prg[2] = prg[1] * prg[1];  prg[3] = x[6];  prg[4] = log(prg[2]);  prg[5] = exp(prg[1]);  prg[6] = prg[0] - prg[5];  prg[7] = x[6];  prg[8] = x[4];  prg[9] = log(prg[7]);  prg[10] = prg[4] * prg[4];  prg[11] = prg[9] + prg[4];  prg[12] = prg[7] / prg[10];  prg[13] = prg[5] * prg[10];  prg[14] = x[1];  prg[15] = x[0];  prg[16] = x[1];  prg[17] = prg[8] * prg[3];  prg[18] = x[4];  prg[19] = log(prg[13]);  prg[20] = prg[19] / prg[7];  prg[21] = x[3];  prg[22] = x[4];  prg[23] = prg[4] / prg[11];  prg[24] = x[3];  prg[25] = prg[3] - prg[12];  prg[26] = x[0];  prg[27] = log(prg[12]);  prg[28] = prg[15] * prg[24];  prg[29] = x[2];  prg[30] = prg[25] - prg[17];  prg[31] = x[0];  prg[32] = x[4];  prg[33] = x[5];  prg[34] = x[5];  prg[35] = x[6];  prg[36] = prg[1] - prg[11];  prg[37] = x[4];  prg[38] = prg[6] - prg[2];  prg[39] = prg[34] - prg[4];  prg[40] = x[6];  prg[41] = prg[23] - prg[17];  prg[42] = prg[20] / prg[30];  prg[43] = prg[9] - prg[20];  prg[44] = x[4];  prg[45] = x[0];  prg[46] = prg[23] + prg[25];  prg[47] = prg[20] + prg[46];  prg[48] = prg[47] + prg[42];  prg[49] = x[0];  outputs[0] = prg[48];  }  int main(void)  {  //example of utilization ...  double x[7];  x[0] = 735.000000;  x[1] = 441.000000;  x[2] = 0.000000;  x[3] = 147.000000;  x[4] = 56.000000;  x[5] = 294.000000;  x[6] = 200.000000;  double outputs[1];  mepx(x, outputs);  printf("%lf", outputs[0]);  getchar();  } |
| --- |

**Table S5.** MEP generated code for compressive strength prediction

| #include <math.h>  #include <stdio.h>  void mepx(double *x /*inputs*/, double *outputs)  {  double prg[50];  prg[0] = x[3];  prg[1] = prg[0] + prg[0];  prg[2] = log(prg[1]);  prg[3] = x[5];  prg[4] = x[6];  prg[5] = log(prg[3]);  prg[6] = log(prg[5]);  prg[7] = log(prg[4]);  prg[8] = log(prg[6]);  prg[9] = log(prg[7]);  prg[10] = x[0];  prg[11] = prg[2] - prg[5];  prg[12] = x[3];  prg[13] = prg[6] * prg[11];  prg[14] = x[6];  prg[15] = x[2];  prg[16] = prg[10] * prg[7];  prg[17] = prg[8] - prg[15];  prg[18] = prg[6] * prg[15];  prg[19] = prg[10] - prg[16];  prg[20] = prg[7] + prg[0];  prg[21] = prg[2] - prg[4];  prg[22] = x[2];  prg[23] = prg[9] + prg[13];  prg[24] = x[0];  prg[25] = x[3];  prg[26] = prg[21] * prg[21];  prg[27] = x[6];  prg[28] = prg[8] - prg[1];  prg[29] = x[1];  prg[30] = exp(prg[17]);  prg[31] = exp(prg[8]);  prg[32] = log(prg[8]);  prg[33] = exp(prg[23]);  prg[34] = x[2];  prg[35] = prg[8] + prg[0];  prg[36] = log(prg[1]);  prg[37] = x[4];  prg[38] = prg[33] - prg[11];  prg[39] = x[6];  prg[40] = x[4];  prg[41] = x[6];  prg[42] = prg[30] + prg[38];  prg[43] = x[3];  prg[44] = x[3];  prg[45] = x[5];  prg[46] = x[6];  prg[47] = x[3];  prg[48] = x[5];  prg[49] = prg[31] + prg[33];  outputs[0] = prg[42];  }  int main(void)  {  //example of utilization ...  double x[7];  x[0] = 875.000000;  x[1] = 875.000000;  x[2] = 0.000000;  x[3] = 162.000000;  x[4] = 18.000000;  x[5] = 152.100000;  x[6] = 7.000000;  double outputs[1];  mepx(x, outputs);  printf("%lf", outputs[0]);  getchar();  } |
| --- |

**Table S6.** MEP generated code for elastic modulus prediction

| #include <math.h>  #include <stdio.h>  void mepx(double *x /*inputs*/, double *outputs)  {  double prg[50];  prg[0] = x[3];  prg[1] = log(prg[0]);  prg[2] = x[2];  prg[3] = x[0];  prg[4] = log(prg[3]);  prg[5] = prg[4] * prg[1];  prg[6] = prg[3] * prg[5];  prg[7] = x[2];  prg[8] = log(prg[7]);  prg[9] = prg[5] - prg[7];  prg[10] = prg[3] + prg[5];  prg[11] = x[5];  prg[12] = x[0];  prg[13] = prg[1] - prg[8];  prg[14] = prg[10] - prg[7];  prg[15] = x[4];  prg[16] = prg[14] - prg[10];  prg[17] = prg[9] - prg[2];  prg[18] = x[0];  prg[19] = x[0];  prg[20] = x[4];  prg[21] = x[4];  prg[22] = exp(prg[9]);  prg[23] = x[4];  prg[24] = x[0];  prg[25] = prg[22] * prg[2];  prg[26] = x[5];  prg[27] = prg[14] + prg[14];  prg[28] = prg[20] - prg[27];  prg[29] = x[0];  prg[30] = prg[4] * prg[14];  prg[31] = prg[6] + prg[30];  prg[32] = prg[4] - prg[14];  prg[33] = x[1];  prg[34] = x[1];  prg[35] = prg[13] * prg[31];  prg[36] = prg[28] + prg[35];  prg[37] = prg[36] - prg[27];  prg[38] = x[6];  prg[39] = prg[37] - prg[27];  prg[40] = prg[39] * prg[14];  prg[41] = prg[20] + prg[39];  prg[42] = x[0];  prg[43] = prg[35] - prg[24];  prg[44] = x[6];  prg[45] = x[3];  prg[46] = prg[45] - prg[37];  prg[47] = prg[31] + prg[7];  prg[48] = prg[39] + prg[28];  prg[49] = prg[48] + prg[25];  outputs[0] = prg[49];  }  int main(void)  {  //example of utilization ...  double x[7];  x[0] = 7.000000;  x[1] = 330.000000;  x[2] = 35.000000;  x[3] = 230.000000;  x[4] = 0.000000;  x[5] = 704.370000;  x[6] = 795.000000;  double outputs[1];  mepx(x, outputs);  printf("%lf", outputs[0]);  getchar();  } |
| --- |
